# A radio oval above Earth’s auroral oval

**DOI:** 10.1126/sciadv.aec4114

**Published:** 2026-03-27

**Authors:** Siyuan Wu, Daniel K. Whiter, Laurent Lamy, Ulrich Taubenschuss, Philippe Zarka, Brieuc Collet, Xiangyu Wang, Georg Fischer, Hao Ning, Yao Chen, Mengmeng Wang, Shengyi Ye, Zhonghua Yao, William S. Kurth, Xiaoli Luan

**Affiliations:** ^1^ School of Physics and Astronomy, University of Southampton, Southampton, UK.; ^2^ Aix Marseille University, CNRS, CNES, LAM, Marseille, France.; ^3^ LIRA, Observatoire de Paris, CNRS, PSL, Sorbonne Université, Université Paris Cité, Meudon, France.; ^4^ Department of Space Physics, Institute of Atmospheric Physics of the Czech Academy of Sciences, Prague, Czech Republic.; ^5^ Planetary Plasma and Atmospheric Research Center, Graduate School of Science, Tohoku University, Sendai, Miyagi, Japan.; ^6^ Shandong Key Laboratory of Space Environment and Exploration Technology, Institute of Space Sciences, Institute of Frontier and Interdisciplinary Science, Shandong University, Weihai, Shandong, China.; ^7^ Institute of Physics, University of Graz, Graz, Austria.; ^8^ Institute of Frontier and Interdisciplinary Science, Shandong University, Qingdao, Shandong, People’s Republic of China.; ^9^ Swedish Institute of Space Physics, Uppsala, Sweden.; ^10^ Department of Earth and Space Sciences, Southern University of Science and Technology, Shenzhen, People’s Republic of China.; ^11^ Department of Earth Sciences, NWU-HKU Joint Centre of Earth and Planetary Sciences, University of Hong Kong, Hong Kong, People’s Republic of China.; ^12^ Department of Physics and Astronomy, University of Iowa, Iowa City, IA, USA.; ^13^ Deep Space Exploration Laboratory/School of Earth and Space Sciences, University of Science and Technology of China, Hefei, Anhui, People’s Republic of China.

## Abstract

Auroral kilometric radiation (AKR), Earth’s strongest radio emission, has long been associated with discrete auroras and electrons near a few kilo–electron volt (keV) range. However, auroras also occur in diffuse forms with broader electron energies, raising the question of why AKR has not been observed above diffuse auroras or linked to electrons outside the kilo–electron volt population. Comprehensive AKR source distributions have remained elusive because of observational limitations, and their local-time coverage remains largely unknown. Using spacecraft measurements, we identify a “radio oval” above the optical auroral oval, spanning the full local-time range, where AKR is emitted over both discrete and diffuse auroras. The AKR source electrons display diverse precipitation features, including monoenergetic (peak flux at 3.82 kilo–electron volts), broadband (1.34 kilo–electron volts), low-energy (0.47 kilo–electron volts), and diffuse types (>1 kilo–electron volt). These results reveal that the cyclotron maser instability—the mechanism driving AKR—can arise in diverse plasma environments, broadening our understanding of both AKR generation and auroral complexity.

## Teaser

Radio waves called AKR radiate above auroras, driven by diverse electron acceleration and precipitation processes.

## INTRODUCTION

Auroral kilometric radiation (AKR) is a nonthermal radio emission generated in Earth’s polar magnetosphere along auroral magnetic field lines (*1*, *2*), where energetic electron precipitation occurs, and the aurora display is generated at the bottom of these field lines at ionospheric altitudes. AKR is generated via the electron cyclotron maser instability [CMI; (*3*, *4*)], a relativistic resonance between auroral electrons and electromagnetic waves near the local electron cyclotron frequency, *fce* ≈ *28* × *B*, with magnetic field *B* in nanotesla and *fce* in hertz. AKR emissions typically span 50 to 800 kHz, peak between 200 and 400 kHz, and originate along magnetic field lines at geocentric distances of ~1.2 to 3.7 Earth radii [Re; (*5*–*7*)].

The AKR source regions—so-called auroral cavities characterized by strong magnetic fields and low plasma densities—have been directly sampled by spacecraft such as Viking, Freja, and Fast Auroral SnapshoT Explorer (FAST) (*8*–*12*). Within these cavities, CMI amplifies electromagnetic waves when the electron distribution function (EDF) exhibits a population inversion, typically manifested as strong perpendicular gradients in the velocity space (*3*, *13*, *14*). A variety of nonthermal EDFs can satisfy this condition, most notably shell-type and loss cone–type distributions (*4*). Shell-type distributions favor perpendicular wave propagation and produce emissions slightly below the local *fce*, while loss cone–type distributions support oblique propagation and generate emissions slightly above *fce* (see Materials and Methods) (*4*, *15*, *16*). Electron measurements reveal that the AKR source EDFs are predominantly of the shell-type horseshoe distribution—formed through parallel acceleration and magnetic mirroring of the electrons and characterized by a shell structure with an embedded loss cone (*12*, *17*–*19*).

AKR-producing electrons typically have kilo–electron volt energies. In time-energy spectrograms, these populations appear as narrow, monoenergetic peaks that form characteristic “inverted-V” signatures (*5*, *12*), which are widely interpreted as the result of quasistatic acceleration by field-aligned potential drops. These signatures are closely associated with discrete auroral arcs (*20*–*22*). A substantial body of observational evidence has established a strong correlation between AKR emissions, monoenergetic electron precipitation, and discrete auroral structures (*2*, *23*, *24*).

Beyond quasistatic acceleration, shell-type EDFs can also be generated by dispersive Alfvén waves (*18*, *19*, *25*, *26*), which energize electrons over a broad energy range via wave-parallel electric fields (*27*). Unlike monoenergetic structures, these events produce broadband-energy electron precipitation, which generates Alfvénic aurora—also observed in discrete morphological forms (*21*, *28*)—demonstrating that AKR can be generated under Alfvénic acceleration conditions.

A third category of precipitation—termed “nonaccelerated”—arises from pitch-angle scattering of plasma sheet electrons, spanning energies from a few electron volts to tens of kilo–electron volts (*29*), via wave-particle interactions between electrons and plasma waves such as whistler-mode chorus and electron cyclotron harmonic waves (*30*–*34*). These interactions drive diffuse aurora (*35*, *36*). Notably, at Earth, AKR has not been linked to diffuse auroral precipitation, contrasting with recent observations at Jupiter where CMI emissions are associated with regions magnetically connected to diffuse aurora (*14*). Furthermore, although auroral electrons span a broad energy range through particle acceleration and precipitation processes [~100 eV to >100 keV; (*21*, *37*)], AKR has traditionally been attributed primarily to electrons of several kilo–electron volts associated with discrete aurora (*2*, *12*). However, recent in situ and remote observations at Jupiter reveal CMI emissions driven by electrons ranging from subkilo–electron volts to more than 20 keV (*26*, *38*), challenging conventional views on the energy thresholds required for CMI and motivating a reassessment of the electron populations capable of generating AKR.

The global distribution of AKR source regions also remains poorly characterized. Prior studies suggest that AKR is confined to limited sectors of the auroral oval, yet comprehensive mappings remain lacking (*39*–*41*). Direct imaging of AKR sources is currently infeasible because of their long wavelengths (approximately kilometers), which far exceed typical spacecraft antenna lengths [e.g., the electric antennas used in this study on the Polar spacecraft are dipole/monopole antennas with lengths of 100 to 130 m; (*42*)]. Consequently, source localization has typically relied on direction-finding techniques (*40*, *43*) or multispacecraft triangulation methods (*41*, *44*). Direction-finding approaches often assume straight-line propagation, an assumption likely invalid near the emission source. To address this, previous studies (*44*, *45*) developed a tangent-plane model for AKR beaming, confining most ray paths within 15° of the tangent plane and explaining the strong upward directionality caused by radio wave refraction as rays exit the auroral cavity. Instead, we use in situ detection, which remains the most precise means of localizing AKR sources (*23*, *46*), although such observations are limited by spacecraft orbital geometry. The Polar spacecraft, with its highly inclined orbit, frequently traversed southern hemisphere AKR source regions at radial distances of ~1 to 2 Re, providing a unique opportunity for direct, high-accuracy AKR source measurements.

Here, we present a systematic analysis of in situ AKR source crossings observed by the Polar spacecraft, examining their spatial correlation with auroral morphology and precipitating electron populations. We demonstrate that AKR originates from a distinct “radio oval” colocated with the auroral oval in geomagnetic latitude and local time but located at higher altitudes than optical emission. Our findings reveal that AKR is generated not only above discrete auroras but also in regions linked to diffuse precipitation. The diversity of associated electron populations indicates that multiple acceleration mechanisms—including quasistatic potential drops, Alfvénic processes, and wave-particle interactions—collectively establish the conditions required for CMI, providing not only a comprehensive framework for understanding radio emission generation in Earth’s magnetosphere but also a unifying perspective on similar processes operating at other magnetized planets and, potentially, at exoplanets.

## RESULTS

### Identification of AKR source crossings and associated plasma conditions

**Fig. 1.**
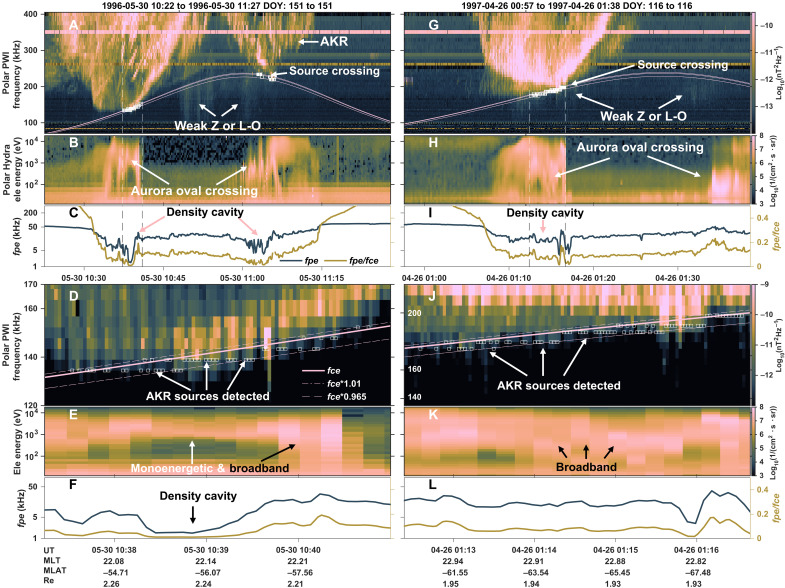
Polar observations of AKR source crossings. (**A**) Spectrogram of the wave magnetic component measured by Polar PWI, with wave intensity color coded as a function of time and frequency. White squares indicate the locations of identified AKR source crossings. Solid, dashed, and dot-dashed pink lines mark the local electron cyclotron frequency (*fce*), 0.965 × *fce*, and 1.01 × *fce* (almost overlap with the solid pink *fce* line), respectively. (**B**) Omnidirectional electron differential energy flux measured by the Hydra instrument. (**C**) Electron plasma frequency (dark blue) calculated from the EFI and the ratio between electron plasma frequency and electron cyclotron frequency (brown). Vertical dashed lines in (A) to (C) indicate the time interval expanded in (D) to (F). Panels (**D**) to (**F**) have the same format as (A) to (C), showing a zoomed-in view of the selected time window. (**G** to **L**) Second example of AKR source region crossing, presented in the same format as (A) to (F).

To identify AKR source regions, we applied an automated detection algorithm to data from the Polar spacecraft. The Plasma Wave Investigation (PWI) instrument (*47*), which operated from March 1996 to September 1997, provided high-resolution AKR wave spectrograms. Source crossings were defined as AKR emissions occurring within a narrow frequency window from 96.5 to 101% of the local *fce*. The lower bound (96.5% *fce*) excludes Earthward-propagating emissions that may cross *fce* but are not locally generated (see Fig. 1). Emissions in this sub-*fce* range are reliably attributed to shell-type electron distributions, known to drive CMI growth slightly below *fce* (*4*). In contrast, emissions slightly above *fce* are more ambiguous and may originate from non–shell-type distributions [e.g., loss cone or conic; (*14*, *48*)] or remote sources observed after propagation. Without in situ measurements of the particle EDF and the corresponding growth rate calculations, the source of these emissions slightly above *fce* cannot be definitively confirmed. However, analogous emissions just above *fce* have been observed at Jupiter, linked to loss-cone and conic distributions (*14*, *38*). To avoid excluding potential source events, we retain these “possible” crossings in our analysis and interpret them as emissions driven by loss-cone distributions. On the basis of wave-particle resonance conditions (see Eqs. 1 and 2 in Materials and Methods), the theoretical resonant electron energies associated with emissions in the 96.5 to 101% *fce* window range from 0 (100% *fce*) to ~18.5 keV (96.5% *fce*) for shell-type EDFs and from 0 (100% *fce*) to ~5.2 keV (101% *fce*) for loss cone–type EDFs.

Figure 1A presents a representative AKR spectrogram obtained by Polar/PWI near a source region. White squares denote the automatically identified source locations, which cluster around the *fce* line (solid pink) and fall within the 96.5 to 101% *fce* bounds (dot-dashed and dashed pink lines, which closely overlap the *fce* line, calculated using the magnetic field obtained from the MFE (Magnetic Fields Experiment) instrument onboard Polar (*49*). During this interval, Polar was situated in the southern polar magnetosphere. Coincident with these AKR detections, elevated electron fluxes observed by the Hydra instrument (*50*) in Fig. 1B indicate crossings of the auroral oval, reinforcing the well-established association between AKR and aurora (*2*, *23*, *46*). Plasma parameters measured within these source regions (Fig. 1C) exhibit conditions favorable for the CMI. Specifically, the plasma is strongly magnetized (*fce* > 100 kHz; pink line), while the electron plasma frequency, *fpe* ≈ 8980*$\sqrt{n}$, with *n* as the electron number density in cm^−3^ and *fpe* in Hz, remains lower (<~20 kHz). The electron number density is obtained from the EFI (Electric Fields Instrument) (*51*). This yields *fpe*/*fce* ratios (brown line) consistently below 0.2, slightly higher than the previously reported threshold of ~0.14 in the AKR source region (*8*) yet still well within the range supporting CMI growth (*4*).

**Fig. 2.**
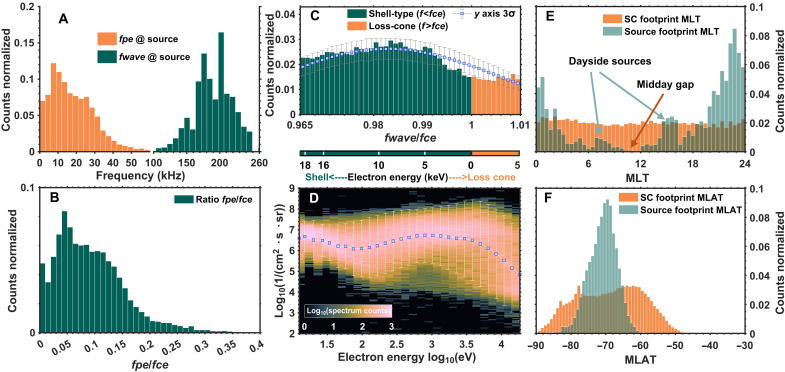
Statistical characteristics of AKR source regions. (**A**) Distributions of electron plasma frequency (orange histogram) and wave frequency (dark green histogram) at AKR source crossings detected by Polar. (**B**) Histogram of the ratio between the electron cyclotron frequency (*fce*) and the electron plasma frequency (*fpe*) in the AKR source regions. (**C**) Distribution of wave frequency normalized to the local electron cyclotron frequency (*fwave*/*fce*). The green histogram corresponds to sources with wave frequencies below *fce*, indicative of shell-type electron distributions; orange histogram corresponds to sources with frequencies above *fce*, suggestive of loss cone–type distributions. The equivalent source electron energies inferred from these ratios are shown along the lower second *x* axis. Error bars denote the average and three standard deviation range for each histogram bin, derived from the instrument’s frequency resolution and 1000 Monte Carlo realizations with random Gaussian noise (see Materials and Methods for details). (**D**) Superposed electron energy spectra observed at AKR source crossings. The electron spectra (electron differential energy flux as a function of energy) recorded during all AKR source crossings are superposed. The color scale (logarithmic) indicates the occurrence frequency as a function of energy and differential energy flux. The mean spectrum is shown as blue squares, with vertical white error bars representing the ±3σ range. (**E**) MLT distribution of AKR source footprints (green) and distribution of Polar spacecraft footprints (orange), limited to times when the local electron cyclotron frequency was within 100 to 260 kHz [see also (A)]. Footprints are mapped from spacecraft altitude to 130 km using the Tsyganenko T89 model. (**F**) Same format as (E) but showing the MLAT distribution. SC, spacecraft.

Emissions generated at frequencies slightly above *fce* are typically linked to loss cone–type electron distributions, although confirming this observationally is challenging, whereas shell-type distributions generally produce emissions below *fce*, as discussed above. A zoomed-in view of the spectrogram (Fig. 1D) reveals both above- and below-*fce* emission sources, indicating the simultaneous presence of both electron populations. However, because of the ~3% frequency resolution of the Polar/PWI instrument relative to the local *fce*, most AKR signals occupy only a single frequency channel near *fce*, limiting our ability to unambiguously classify the source distribution. This limitation is revisited in the context of Fig. 2.

The electron energy spectra from the source region (Fig. 1E) exhibit both monoenergetic and broadband features. Monoenergetic populations are characterized by narrow energy peaks near a few kilo–electron volts, while broadband components span from several hundred electron volts to ~10 keV. Although the measurements shown are omnidirectional because of the lack of field-aligned resolved flux data, this limitation is not expected to substantially affect the interpretation during the aurora oval crossings. A second AKR event, shown in Fig. 1 (G to L), reveals a similar overall structure. However, the electron spectrum in this event (Fig. 1K) displays more dominant broadband features, indicative of stronger Alfvénic acceleration (*52*). The coexistence of monoenergetic and broadband electron precipitation across multiple events suggests that both quasistatic potential drops and Alfvénic processes contribute to the formation of electron distributions that favor CMI and AKR generation (*17*–*19*). In both cases (Fig. 1, D and K), stronger intensities of the emission sources tend to occur in regions of more strongly depleted electron density, where the *fpe*/*fce* ratio is close to zero. In contrast, weaker sources are found in regions with small, although not extreme, *fpe*/*fce* ratios—generally below 0.2. This is further explored in the following sections.

A total of 18,016 AKR source crossings [number of identified pixels on time-frequency plane (with a time resolution of ~2 s), 15,715 sources *fwave* < *fce* and 2394 sources *fwave* > *fce*] were identified by our detection algorithm, with their statistical properties summarized in Fig. 2. The AKR wave frequencies (Fig. 2A, dark green histogram) range from ~100 to 260 kHz, corresponding to source altitudes of ~5000 to ~9000 km when estimated using a simple dipole model. The *fpe* values (orange histogram) are generally below 50 kHz and are mostly under ~30 kHz, i.e., equivalent electron number densities range from 31 cm^−3^ to below 11 cm^−3^.

The *fpe*/*fce* ratio (Fig. 2B) remains predominantly below 0.3. These values are separately examined for shell-type and loss cone–type AKR sources, revealing no notable difference between the two cases (see figs. S1 and S2 and the discussions in the following sections). The distribution in Fig. 2B is broadly consistent with the value of 0.14 reported from Viking measurements (*8*), reflecting the low-density, strongly magnetized plasma conditions required for CMI generation. Notably, the presence of relatively larger *fpe*/*fce* values provides additional insight, as this ratio plays a critical role in determining the dominant growth wave modes, as well as the excitation of harmonic emissions. For instance, the loss cone–driven CMI produces dominant right-hand extraordinary mode (R-X) emissions and weak first harmonic also in R-X mode when *fpe*/*fce* < 0.3. When 0.3 < *fpe*/*fce* < 1, the growth rates of fundamental left-hand ordinary (L-O) mode and harmonic R-X mode gradually become dominant over the fundamental X mode intensity (*53*, *54*). However, the absence of polarization measurements prevents us from identifying the wave mode. The relationship between wave intensities observed in the source region and the *fpe*/*fce* ratio is examined in fig. S1, revealing a trend in which stronger emission sources are generally found in regions with smaller *fpe*/*fce* ratios, consistent with the observation in Fig. 1. However, numerous sources with varying intensity levels are also observed under similar *fpe*/*fce* conditions, suggesting that this ratio is not the sole factor influencing wave excitation.

The *fwave*/*fce* ratio (Fig. 2C) falls within 0.965 to 1.01, constrained by our source detection criteria. The resonance energies of the source electrons can be estimated from the frequency offset using Eqs. 1 and 2 (see Materials and Methods). A small peak between 0.980 and 0.995 corresponds to resonance energies of ~3 to 10 keV, generated from shell-type distributions. However, given the ~3% frequency resolution of the Polar/PWI instrument at these altitudes, these estimates carry considerable uncertainty, as reflected by the error bars above each histogram bin. The distribution can still provide finer information within this 3% resolution because the local *fce*, derived from magnetometer measurements, varies at a much finer scale. The typical uncertainty in *B* is ~1 nT, corresponding to ~28 Hz in *fce*, which allows a much more precise determination of *fwave*/*fce* than the wave instrument’s frequency resolution alone. For a single detected source pixel in the time-frequency plane, a ~3% frequency resolution corresponds to an error of up to ±1.5% within one frequency channel, which translates to energy uncertainties of ~7.78 keV for shell distributions and ~7.85 keV for loss-cone distributions, as estimated from *fwave*/*fce* using Eqs. 1 and 2 (see Materials and Methods). To quantify this effect, we calculated the mean and standard deviation of each histogram bin by adding Gaussian noise to the measured source frequencies, with the noise amplitude drawn from a normal distribution and scaled by half of the instrument’s frequency resolution. This procedure was repeated 1000 times, yielding substantial uncertainties but still supporting a possible peak between 0.980 and 0.995, as reflected in the average values (see Materials and Methods for details).

Figure 2D shows a superposed analysis of electron energy spectra at AKR source crossings. Specifically, all electron spectra measured during the identified AKR source crossings are stacked together to obtain an average energy spectrum representative of the source region. The blue-squared white line represents the mean spectrum, with white error bars indicating the ±3σ flux range. Although on the basis of omnidirectional fluxes, the averaged spectrum reveals broad enhancement at higher energies (~100 eV to 10 keV), consistent with a bump-on-tail (or population inversion) feature capable of providing the free energy required to drive the CMI. These energy spectra are further categorized by precipitation type in the following sections.

The AKR source footprints, traced to auroral altitudes [~130 km, consistent with the typical emission height of auroral features observed by the Polar Ultraviolet Imager (UVI); (*55*)], are shown in Fig. 2E [magnetic local time (MLT)] and Fig. 2F [magnetic latitude (MLAT)], which are expressed in Altitude-Adjusted Corrected Geomagnetic (AACGM2) coordinates, a commonly used system for transforming ground-based and space-based measurements into magnetic coordinates (*56*). Magnetic mapping is performed using the T89 magnetic field model (*57*). For comparison, Polar’s orbital coverage (orange histograms) is also plotted for the regions with *fce* between 100 and 260 kHz, consistent with the AKR source altitudes. The relatively uniform orbital coverage confirms that the observed peaks in source occurrence are not artifacts of observational bias. The MLT distribution shows a clear preference for the nightside, particularly in the premidnight sector. Additional peaks near MLT = 6 to 9 and MLT = 14 to 16 are observed, and they correspond to the “hot spot” and “warm spot” dayside auroral regions, respectively (*58*–*60*). The MLAT distribution is concentrated between around −60° and −80°, consistent with the typical boundaries of the auroral oval (*58*). Note that all AKR source crossings are observed in the southern hemisphere, which is attributed to the difference in Polar’s orbital altitude between the two hemispheres.

### AKR radio oval and its correspondence with the auroral oval

**Fig. 3.**
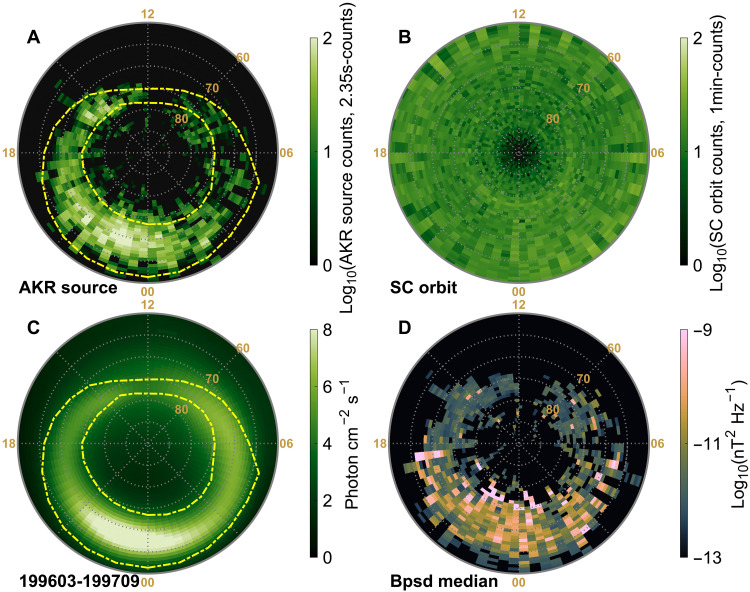
AKR radio oval and auroral oval comparison. (**A**) Footprints of AKR sources projected to auroral altitudes (130 km) in the AACGM2 coordinate system (*56*) in the southern hemisphere, color coded by the AKR source count detected by Polar. The yellow dashed lines indicate the statistical auroral oval model from a previous work (*61*), plotted for *Kp* = 3. (**B**) Footprints of the Polar spacecraft during intervals when the local electron cyclotron frequency was within the 100 to 260 kHz range. (**C**) Synoptic northern auroral oval compiled from Polar UVI observations between March 1996 and September 1997. (**D**) Median AKR wave intensity distribution, color-coded by median magnetic wave intensity. Bpsd, wave magnetic field power spectral density.

AKR source footprints were projected onto magnetic polar coordinates (MLT versus MLAT in AACGM2 coordinates) with the size of each bin being ^1^/_3_-hour × 1° MLAT, as shown in Fig. 3A. For comparison, the statistical auroral oval boundary from a previous work (*61*) at *Kp* = 3 is overlaid as a yellow dashed line, which is consistent with the typical geomagnetic conditions (*Kp* ≈ 1 to 3) during the observed AKR source crossings. The corresponding Polar spacecraft footprint coverage, limited to regions where *fce* ranges from 100 to 260 kHz, is shown in Fig. 3B. The uniform distribution of spacecraft coverage across MLT (also supported by Fig. 2) confirms again that the observed AKR source distribution is not affected by orbital bias.

To directly compare with auroral morphology, Fig. 3C shows the averaged global auroral emission map derived from Polar UVI observations. This map was generated for the northern hemisphere using auroral images acquired between March 1996 and September 1997, corresponding to the same overall interval as the Polar PWI wave data analyzed in this study. Despite hemispheric differences, this serves as a reliable proxy for the large-scale structure of the auroral oval. The southern AKR source distribution shows a notable resemblance to the northern auroral oval, with AKR sources spanning almost all local times and MLATs typically associated with auroral activity. The highest occurrence is observed in the premidnight sector, consistent with enhanced auroral activity, while a clear reduction of detected AKR sources near MLT ≈ 11:00 aligns with the well-known midday gap (*62*). Additional AKR source peaks near MLT = 9 and MLT = 15 correspond to the dayside “hot spot” and “warm spot” auroras (*59*, *60*), which exhibit strong seasonal variation and may reflect the seasonal behavior of AKR, as suggested here—warranting further targeted analysis. On the poleward side of the AKR radio oval, we also identify source regions at latitudes extending beyond the model auroral oval and the approximate poleward edge of the auroral emissions in Fig. 3C. These high-latitude AKR sources may reflect particle precipitation or auroral features inside the polar cap, such as the recently reported link between AKR and high-latitude dayside aurora (*63*). They also recall earlier reports of AKR source crossings along transpolar auroral arcs (*64*). Such events highlight a potentially important connection between AKR and polar-cap auroral phenomena, warranting further investigation.

The spatial coincidence between AKR sources and auroral emissions confirms that AKR is generated throughout the auroral oval. We therefore define this global AKR source distribution as the AKR radio oval—a radio-frequency counterpart to the optical auroral oval. These radio-optical emission connections are reminiscent of similar findings at Saturn, where Saturn’s kilometric radiation sources map precisely to the ultraviolet auroral oval (*65*). At Jupiter, although large-scale spatial correspondence exists between radio and auroral emissions, the radio sources exhibit more diversity: Broadband kilometric emissions tend to coincide with the main aurora (*66*), while hectometric and decametric emission sources are generally associated with the equatorward edge of the oval or with the diffuse aurora (*38*, *67*).

Figure 3D shows the median AKR intensities within each spatial bin. Although emissions occur on both nightside and dayside sectors, dayside AKR is generally weaker, likely reflecting reduced wave growth efficiency as a result of variations in source conditions such as local *fpe*/*fce* ratios and electron precipitation characteristics. To investigate this, fig. S3 compares *fpe*/*fce* and *fwave*/*fce* parameters across different MLTs. No notable difference in *fpe*/*fce* is observed between dayside and nightside sources, although a slight increase appears when the AKR source is near the equatorward edge of the radio oval. Despite frequency resolution limitations, the *fwave*/*fce* parameter suggests that dayside sources may be more closely associated with low-energy electron precipitation than nightside sources (especially for the MLT = ~15:00 AKR sources and for emissions at *fwave* < *fce*), as *fwave*/*fce* values tend to approach 1 (see Eqs. 1 and 2 in Materials and Methods). Consequently, the weaker AKR intensity observed on the dayside may result from low-energy electron precipitation. This potential connection is further explored in subsequent sections.

The spatial distributions of the radio ovals, categorized by emission frequency—above and below *fce* (see fig. S1)—are largely similar. As expected, the intensity of the *fwave* > *fce* oval (loss cone type) is weaker than that of the *fwave* < *fce* oval (shell type), consistent with theoretical predictions that loss cone–driven CMI typically yields lower growth rates than shell-type distributions (*4*). However, as discussed above, the identification of these *fwave* > *fce* sources remains ambiguous.

### Four types of electron features in the AKR source region

**Fig. 4.**
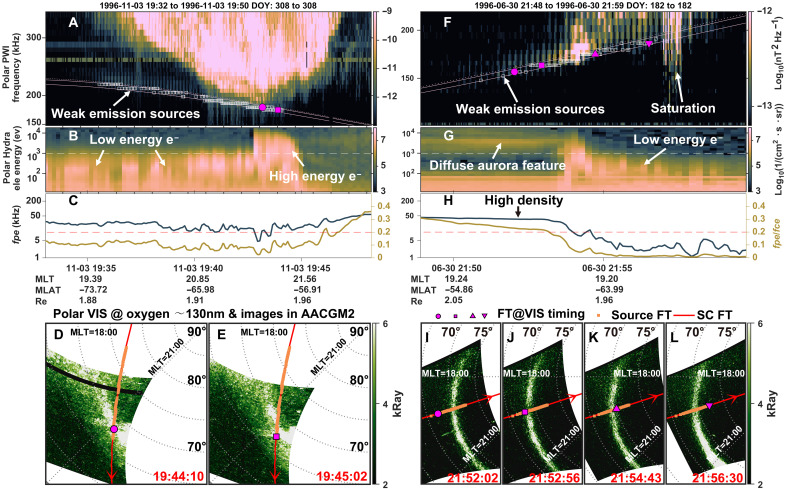
Four types of electron features associated with AKR source regions. (**A**) Polar PWI wave magnetic field spectrogram. Detected AKR sources are indicated by white squares. The pink lines represent the local electron cyclotron frequency: solid for 1.00 *fce*, dashed for 0.965 *fce*, and dotted for 1.01 *fce* (which nearly overlap). (**B**) Omnidirectional electron differential energy flux from Polar Hydra. The white dashed line marks the 1-keV energy level. (**C**) Simultaneous measurements of *fpe* and *fpe*/*fce* ratios. (**D** and **E**) Polar VIS auroral images near the atomic oxygen emission line (~130 nm), taken at 19:44:10 UT and 19:45:02 UT, respectively. The red lines indicate the magnetic footprints of the Polar spacecraft. AKR source footprints are shown as yellow squares. Pink markers denote the closest spacecraft footprint timings corresponding to the auroral image snapshots. (**F** to **L**) Second example of Polar observations within an AKR source region, presented in the same format as (A) to (E). FT, footprint.

Beyond the global perspective of the AKR radio oval, individual source crossing cases allow for a more detailed comparison between AKR sources, electron precipitation, and auroral emissions. Two representative events are shown in Fig. 4. In the first event (Fig. 4, A to E), the Polar spacecraft traversed the southern hemisphere polar cap from dayside to nightside. Initially, weak AKR sources were detected at higher *fce* (Fig. 4A), accompanied by low-energy (<1 keV) electrons (Fig. 4B). As *fce* decreased, stronger AKR sources were encountered, associated with higher-energy (>1 keV) electrons. During this observation, Polar was located within a large-scale auroral cavity characterized by low electron density and an *fpe*/*fce* ratio well below 0.2 (Fig. 4C). The footprints of these strong AKR sources, as well as the high-energy electrons (indicated by pink markers in Fig. 4, A, D, and E), aligned with a bright auroral arc, whereas the weaker emissions and low-energy electron population coincided with its poleward edge. Auroral images were obtained by the Visible Imaging System (VIS) Earth Camera onboard the Polar spacecraft, observing the oxygen emission line near 130 nm (*68*). These observations provide direct evidence that AKR emissions can be driven by electrons with energies below 1 keV, echoing recent findings from Jupiter where CMI emissions can also be driven by subkilo–electron volt electrons (*26*, *38*). Three additional examples of AKR source crossings linked to such low-energy electrons are presented in fig. S4.

The second event (Fig. 4, F to L) captures AKR source crossings during a southern polar cap crossing from duskside to dawnside. Initially, weak AKR sources were accompanied by high-energy but low-flux electrons—a characteristic of diffuse aurora (*21*, *69*)—located equatorward of a bright auroral arc (Fig. 4I, pink circle) (*36*). These diffuse auroral features are faint or invisible in images taken by the VIS, likely due to low emission intensity and instrument sensitivity. As Polar crossed into the brighter part of the aurora, a short interval of enhanced electron flux coincided with stronger AKR emissions. Last, at the poleward edge of the bright arc, low-energy electrons reappeared along with weaker AKR emission sources, echoing the pattern observed in the first event. This observation directly confirmed the relationship of the AKR to the diffuse aurora, albeit at weak emission intensities. The *fpe*/*fce* ratio in Fig. 4H shows that during the diffuse aurora crossing, Polar was outside the auroral cavity (although the density remained low), with *fpe*/*fce* > 0.2 in many cases (see also fig. S5 for additional examples of diffuse aurora-AKR observations). Such diffuse aurora–related AKR, exhibiting a large *fpe*/*fce* ratio—often, although not always (see the *fpe*/*fce* ratios for diffuse aurora-AKR in fig. S2)—suggests distinct plasma parameters for diffuse precipitation–driven emissions, which may contribute to their weaker intensities, as seen in Fig. 1 and further explored in fig. S1, where stronger emissions correlate with much lower *fpe*/*fce* values.

Given that these different types of electron features are associated with either weaker or stronger AKR emissions, and their footprints correspond to different auroral forms and locations relative to the bright aurora arc, we first performed a visual classification of the observed electron spectra in the AKR source regions. This revealed that most observed spectra fall into four types: Type A, Broadband spectra, likely associated with Alfvénic acceleration (see examples in Fig. 1). Type B, Monoenergetic spectra, typically linked to quasistatic potential drops (see examples in Fig. 1). Type C, Low-energy spectra (with peak flux energy <1 keV), possibly linked to weak potential drop or Alfvénic acceleration (see examples in Fig. 4 and fig. S4). Type D, Diffuse aurora–type spectra, showing a low flux peak at high energies (>1 keV), corresponding to unaccelerated electron populations from the plasma sheet (see examples in Fig. 4 and fig. S5).

In previous work (*21*, *22*), monoenergetic and broadband electron precipitations were primarily classified on the basis of the width of the energy peak in the electron spectrum. Using 1-s Defense Meteorological Satellite Program measurements at an altitude of ~850 km (*21*, *22*), a spectrum was identified as monoenergetic if the peak differential energy flux exceeded a defined threshold, was confined to a single energy channel, and fell to ≤30% of the peak value within the two adjacent channels. Spectra were classified as broadband when three or more consecutive channels exceeded the threshold, while all other spectra were categorized as diffuse. However, because our measurements differ from previous studies in both temporal resolution (14-s Polar Hydra electron spectra) and sampling altitude (~5000 to 9000 km), these peak-width criteria cannot be applied directly. We therefore classify spectra on the basis of their morphological characteristics using an automated clustering algorithm (see Materials and Methods). Low-energy spectra could, depending on peak width, be further subdivided into monoenergetic or broadband types; however, in our observations, they generally correspond to weak AKR sources, particularly on the dayside (see discussion below). For this reason, we retain them as a distinct class rather than merging them with monoenergetic or broadband spectra.

### Four types of electron features in the AKR source region and their auroral counterparts

**Fig. 5.**
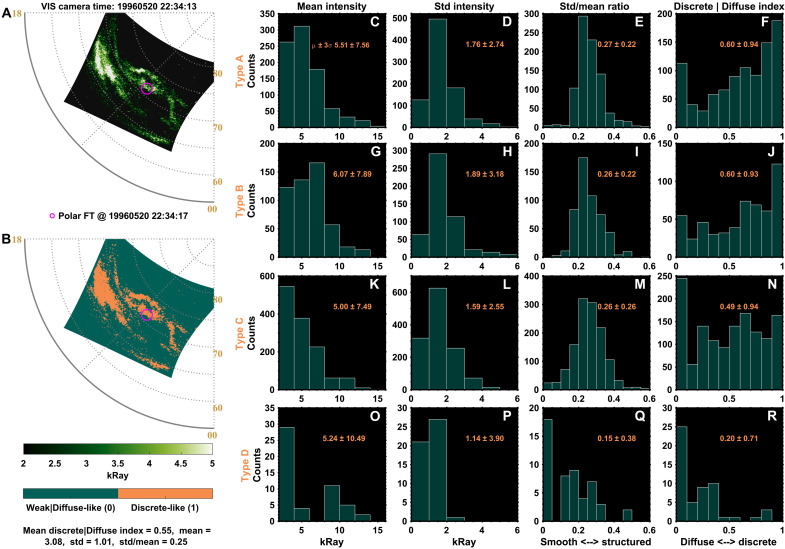
Aurora features associated with the four types of electron spectra. (**A**) Example of a Polar VIS auroral image taken on 20 May 1996 at 22:34:17 UT. The Polar spacecraft footprint at this time is marked by the pink circle. (**B**) Identified discrete auroral boundaries (orange; assigned a value of 1), with weak or diffuse aurora marked as 0 (dark green). (**C**) Average (mean) auroral intensity near the spacecraft footprint, calculated using the timings of type A electron spectra. (**D**) Standard deviation of the auroral intensity near the spacecraft footprint for type A spectra. (**E**) Ratio of standard deviation to the mean auroral intensity. Larger values indicate more structured aurora, while smaller values imply smoother variations. (**F**) Discrete-diffuse index derived from the discrete aurora detection algorithms as shown in (B), where larger values indicate auroral features closer to discrete auroral boundaries and smaller values correspond to weak or diffuse aurora near the footprint. Panels (**G**) to (**J**), (**K**) to (**N**), and (**O**) to (**R**) show the corresponding parameters for type B, type C, and type D electron spectra, respectively, in the same format as (C) to (F).

To examine the auroral morphology associated with each electron spectral type, we extracted auroral parameters from Polar VIS images within ±10 s of each AKR source crossing. Discrete auroral boundaries were identified using an image-based classification algorithm that incorporates auroral intensity, local standard deviation, and intensity gradients, supplemented by visual inspection (see Materials and Methods). Figure 5A shows an example VIS image, and Fig. 5B displays the corresponding discrete-aurora detection output, where discrete regions are labeled as 1 (orange) and all other regions as 0 (dark green). Auroral parameters—including average intensity, standard deviation, the ratio of standard deviation to mean (std/mean), and a discrete-diffuse index—were calculated within a circular area [~1° MLAT radius, shown as a pink circle in Fig. 5 (A and B)] centered at the spacecraft footprint for each spectral type. The discrete-diffuse index is defined as the mean value of the 0-1 detection map in Fig. 5B such that 1 represents discrete aurora and 0 represents diffuse aurora. The resulting auroral parameters are presented in Fig. 5 (C to F) (type A), Fig. 5 (G to J) (type B), Fig. 5 (K to N) (type C), and Fig. 5 (O to R) (type D).

Broadband (type A) and monoenergetic (type B) spectra correspond to strong auroral intensities (Fig. 5, C and G), large standard deviations (Fig. 5, D and H), and high std/mean ratios (Fig. 5, E and I), indicative of highly structured, discrete aurora. The discrete-diffuse index further confirms that type A and type B source crossings are predominantly discrete, with mean values around 0.6 and distributions peaking near 1.

For type C spectra, the auroral intensity is weaker (Fig. 5K), but the standard deviation and std/mean ratio still suggest structured morphology similar to types A and B. The mean discrete-diffuse index (~0.49) reflects this trend, although many events exhibit values near zero. This arises because the precipitation energy and flux are low; in numerous cases, the VIS instrument does not detect clear aurora structures. Consequently, the index distribution shows a low-value peak, reflecting nondetection rather than an absence of structuring.

Type D spectra are associated with weak auroral intensity, the lowest standard deviation and std/mean values, and a near-diffuse index, consistent with smooth auroral forms (Fig. 4). These findings align with previous studies (*21*, *22*, *27*–*29*), indicating that type A and type B spectra correspond to discrete aurora, type C spectra are likely associated with discrete aurora, and type D spectra correspond to diffuse aurora. Some type A or type B events exhibit indices below 0.5, whereas some type D events show indices above 0.5. This discrepancy arises from differences in instrument temporal resolution: Electron spectra are recorded at cadence of ~14 s, while auroral mapping and field-line tracing are performed at ~1-s resolution. Consequently, the spacecraft footprint may traverse weaker auroral regions that are unresolved in the electron spectra. The average auroral intensity alone does not unambiguously indicate auroral form; rather, the standard deviation, std/mean ratio, and discrete-diffuse index provide the primary evidence. Nonetheless, the intensity distributions remain broadly consistent with electron peak flux and AKR intensity patterns, as discussed later.

### Four types of electron features in the AKR source region and their AKR sources

**Fig. 6.**
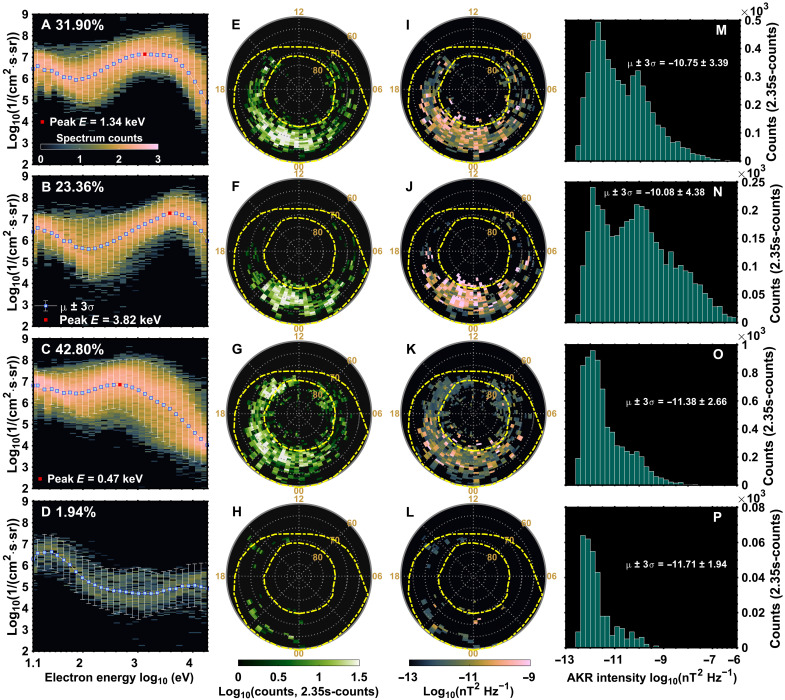
Statistical characteristics of four types of electron spectra and their associated AKR radio oval distributions. (**A** to **D**) Representative electron energy spectra for the four spectra types: (A) broadband, (B) monoenergetic, (C) low energy, and (D) diffuse type. The background color denotes the occurrence frequency as a function of electron energy and differential flux. Blue squares indicate the mean spectrum for each category, and white vertical bars represent the 3σ range. The percentage of occurrence for each type is shown in the upper-left corner of each panel. (**E** to **H**) Spatial distribution of AKR radio ovals corresponding to the four types of electron spectra. The color indicates the number of detected AKR sources. (**I** to **L**) Median AKR wave intensity distributions associated with each spectrum type, mapped over the radio oval. (**M** to **P**) Histograms of AKR wave intensity for each electron spectrum type.

While these spectral features are commonly linked to different acceleration processes, no single acceleration mechanism can be unambiguously assigned to each type as noted previously (*35*). While the broadband and monoenergetic types are more clearly linked to specific mechanisms, the low-energy spectra may result from weak acceleration by a combination of processes: either weak potential drops, Alfvénic acceleration, or both (*35*). Distinguishing between them requires more complicated criteria using particle pitch-angle distributions, which are not available here. As previously shown (*35*), most aurora electron spectra arise from the superposition of multiple acceleration mechanisms acting simultaneously. Hence, the categorized types of different spectra are only an average picture of the physics that may dominate these spectra. The different types of electron spectra were mapped across the AKR radio oval (Fig. 6), illustrating their connection to AKR emissions and the corresponding auroral features shown in Fig. 5.

Figure 6 (A to D) shows the typical spectra for each category. Broadband electrons (Fig. 6A) account for 31.9% of events, with a broad flux enhancement at higher electron energy and a peak flux around 1.34 keV and energies extending up to ~10 keV. Monoenergetic electrons (Fig. 6B) comprise 23.36%, peaking near 3.82 keV with a relatively narrow energy peak at higher energy. Low-energy electrons (Fig. 6C) form the largest group (42.8%), peaking at ~0.47 keV. Diffuse aurora–type electrons (Fig. 6D) are the least common (1.94%), and the spectra generally show a low-flux peak at energies above 1 keV.

Figure 6 (E to H) presents the spatial distributions (“radio ovals”) of each electron spectrum type. Broadband (Fig. 6E) and low-energy spectra (Fig. 6G) are distributed across the full oval. The monoenergetic type (Fig. 6F) is concentrated in the midnight sector, while the diffuse type (Fig. 6H) clusters near the equatorward edge of the auroral oval, primarily in the afternoon and premidnight sectors. The broadband radio oval (Fig. 6E) aligns with the previously identified broadband auroral oval associated with strong solar wind driving conditions (*21*). In contrast, the monoenergetic radio oval (Fig. 6F) is more consistent with the monoenergetic auroral oval observed under weak solar wind driving (*21*). The low-energy spectra pattern (Fig. 6G) likely reflects a combination of both broadband and monoenergetic components, thus offering a limited diagnostic value. Nevertheless, dayside AKR sources—particularly those near MLT = 15—appear to predominantly originate from low-energy precipitation, consistent with the *fwave*/*fce* distribution shown in fig. S3. Precipitating electrons at these energies (~0.47 keV; Fig. 6C) may originate from weakly accelerated magnetospheric populations or from the magnetosheath—both known contributors to dayside auroral activity (*52*, *60*). The diffuse precipitation–type radio oval, while broadly consistent with the notion that diffuse aurora occurs at the equatorward boundary of discrete arcs (*36*), is predominantly observed in the premidnight and afternoon sectors. This contrasts with the expected postmidnight distribution driven by eastward *E* × *B* drift of electrons (*21*). This inconsistency suggests that the formation of CMI-unstable electron distributions may depend on specific scattering processes, potentially involving different plasma wave modes that shape distinct electron populations (*70*).

Figure 6 (I to L) displays the median AKR intensity associated with each type of electron spectrum. Broadband and monoenergetic sources produce the strongest emissions, especially on the nightside. In contrast, low-energy and diffuse sources generate substantially weaker emissions—typically about an order of magnitude lower, if compared to the first two types. This trend is further confirmed by the full intensity distribution plots (Fig. 6, M to P), which show that broadband and monoenergetic types tend to dominate the intense emissions and also consistent with the aurora intensities shown in Fig. 5. This may explain the lack of reported AKR associated with diffuse aurora, as such emissions likely weaken substantially during propagation away from the source region. All these parameters are also separately analyzed for *fwave* < *fce* and *fwave* > *fce*, as shown in figs. S6 and S7 for reference, and are consistent with the results presented here.

## DISCUSSION

**Fig. 7.**
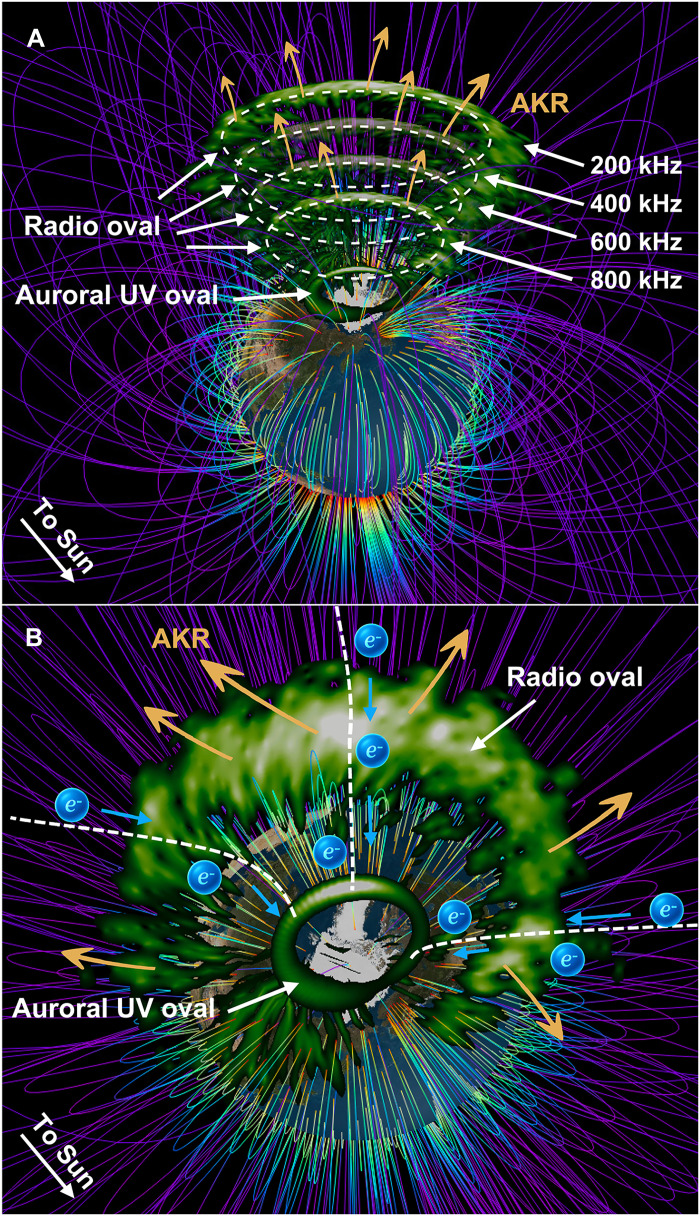
Schematic illustration of the AKR radio oval. (**A**) Side view showing the vertical structure of the AKR radio oval overlaid above the optical auroral oval, with the radio oval represented at four altitudes corresponding to different wave frequencies. Both radio and auroral ovals are adapted and smoothed from Fig. 3 (A and C). (**B**) Top-down view of the AKR radio oval and auroral oval. For clarity, only a single altitude of the radio oval is shown.

This study provides a comprehensive characterization of the AKR source region, which emerges as a “radio oval”—a counterpart to the optical auroral oval that extends to higher altitudes (Fig. 7). As indicated by Fig. 3D, the radio oval is thicker on the nightside, thins toward the dayside, and shows a gap around the noon MLT. The frequency-dependent emission altitude, controlled by the local *fce*, produces a conical or funnel-like structure in space. Global mapping reveals that this radio oval spans both dayside and nightside sectors across a wide range of MLT, with source regions concentrated between 60° and 80° MLAT, peaking near 70°. This large-scale morphology reflects the dynamic coupling between magnetospheric processes and diverse electron acceleration mechanisms that operate across the auroral zones.

An interesting finding is that AKR generation is not limited to monoenergetic electrons accelerated by quasistatic potential drops above discrete aurora or to broadband electrons accelerated by Alfvén waves. Monoenergetic, broadband, low-energy, and diffuse auroral electron spectra all contribute to AKR generation. These distinct types, driven by different mechanisms—such as quasistatic potential drops, Alfvén wave acceleration, and wave-particle scattering—occupy different spatial domains within the radio oval. This allows the radio oval to be subdivided into analogs of optical aurora: broadband radio oval, monoenergetic radio oval, and diffuse-type radio oval. The spatial structure and spectral properties of these emissions thus encode information about the underlying plasma processes and magnetospheric dynamics.

Similar to recent findings at Jupiter—where loss-cone, shell, and conic electron distributions have been shown to drive the CMI (*26*, *67*, *71*–*73*)—our Earth observations, despite lacking direct measurements of EDFs, suggest that multiple electron acceleration and precipitation processes can generate unstable EDFs, at least of the shell type (*fwave* < *fce* sources), which feed the CMI. Furthermore, we provide in situ evidence that AKR generation occurs above diffuse aurora characterized by “nonaccelerated” electron precipitation, likely resulting from pitch-angle scattering of electrons by plasma waves (*30*–*34*). The relatively rare detection of AKR associated with diffuse aurora may be due to the weaker emission intensities observed, potentially explaining why this connection has not been reported in previous studies.

Note that the electron spectra analyzed here are measured in situ at relatively high altitudes (∼5000 to 9000 km), where AKR at 100 to 260 kHz is generated. Below these altitudes, before precipitating into the atmosphere to produce aurora, electrons—particularly those associated with the monoenergetic, broadband, and low-energy types—may undergo additional acceleration. Such further energization is physically plausible because both field-aligned potential drops and inertial Alfvén waves can accelerate electrons along the magnetic field. In this scenario, low-energy populations may evolve into monoenergetic or broadband-like forms at lower altitudes, while initially monoenergetic or broadband electrons may be accelerated to even higher energies. This following acceleration may also contribute to the production of higher-frequency AKR, whose source regions lie at lower altitudes and are expected to involve more energetic and more strongly cavity-depleted electron populations. However, the spectra analyzed here are measured directly within the AKR source region. These distributions therefore represent the electrons immediately responsible for exciting AKR in the observed frequency range, and any additional acceleration that may occur below the source altitude does not influence the conclusions of this work.

These findings carry interesting implications for CMI theory. The detection of source electrons below 1 keV aligns with theoretical predictions that CMI can operate below the kilo–electron volt energy range (*74*) and as also shown by the remote and in situ observations at Jupiter (*14*, *26*, *38*). While R-X mode AKR emissions are typically observed, theoretical work suggests that when the source electron energy is below 1 keV, L-O mode waves may achieve stronger growth (*74*). Furthermore, it has been proposed that when the cold plasma background in the radio source region is not negligible—i.e., the electron density is relatively high, leading to a large *fpe*/*fce* ratio or a large cold-to-hot electron density ratio—the CMI may excite the intermediate, trapped Z mode emissions (*75*, *76*). A substantial portion (see fig. S2) of the diffuse aurora source regions we observed appears to exhibit such elevated *fpe*/*fce* ratios, which may be consistent with the generation of these predicted Z mode waves. Our findings support this possibility, although the lack of polarization measurements precludes a definitive identification of the wave mode.

Electron density measurements indicate that most AKR source regions reside within an auroral cavity characterized by reduced plasma density. Within this large-scale auroral cavity, plasma density is generally low both at auroral field lines—where densities are the lowest—and near the polar cap. Localized regions of further density depletion often appear within this cavity, indicating fine-scale plasma structures. AKR sources are typically observed within this extensive low-density cavity and, in some cases, are associated with these smaller-scale density depletions nested inside the larger cavity (Figs. 1 and 4). This is supported by the observed *fpe*/*fce* ratio, which remains below 0.3 in most cases. When analyzed by electron spectrum type, all types of electron spectra except diffuse-type exhibit systematically low ratios—mostly <0.14—consistent with typical auroral cavity conditions. In contrast, the diffuse aurora shows a broader distribution with a substantial fraction of *fpe*/*fce* > 0.14 (fig. S2).

This unique long-term dataset of AKR source crossings provides a rare opportunity to revisit AKR generation processes in the absence of direct velocity-space EDF measurements. Enabled by the exceptional orbital coverage and instrumentation of the Polar mission, this dataset is unlikely to be replicated in the foreseeable future. In addition to reaffirming the established roles of Alfvénic and quasistatic acceleration, our analysis reveals observational connections to diffuse aurora and low-energy electron precipitation. Together, these findings provide an integrated picture of the conditions that enable CMI-driven radio emission, contributing to a more universal understanding of such processes—not only for AKR at Earth but also for radio emissions at other magnetized planets and even exoplanets.

## MATERIALS AND METHODS

### Automated detection of AKR source crossings

The CMI is a weakly relativistic electron resonance mechanism in which electromagnetic waves are amplified through the relativistically corrected electron cyclotron resonance condition (*3*): $\omega =\frac{\omega_{\text{ce}}}{\Gamma }+k_{∥}v_{∥}$, where $\Gamma =\frac{1}{\sqrt{1-\frac{v^{2}}{c^{2}}}}$ is the Lorentz factor, ω is the wave angular frequency, $\omega_{\text{ce}}=2\pi f_{\text{ce}}$ is the angular electron cyclotron frequency, $k_{∥}$ is the parallel wave vector, and $v_{∥}$ is the parallel velocity of the resonant electrons. This resonance condition describes how the amplified wave frequency corresponds to the relativistically corrected and Doppler-shifted electron cyclotron frequency. In the semirelativistic approximation, the condition can be reorganized into the form $v_{⊥}^{2}+{\left(v_{∥}-v_{0}\right)}^{2}=v_{r}^{2}$, which describes a resonance circle in the velocity space. It is centered at $v_{0}=\frac{k_{∥}c^{2}}{\omega_{\text{ce}}}$, with a radius of the circle: $v_{r}=\sqrt{v_{0}^{2}-2c^{2}∆\omega }$, where $∆\omega =\frac{\omega -\omega_{\text{ce}}}{\omega_{\text{ce}}}$. The wave growth rate is then obtained by integrating the gradient of the EDF *f* in the electron velocity phase space, $\frac{\partial f\left(v_{∥},v_{⊥}\right)}{\partial v_{⊥}}$, along this resonance circle. In practice, for a given EDF, the true wave growth rate corresponds to the maximum growth rate obtained by evaluating the CMI growth along a set of resonance circles with varying centers and radii. This approach effectively identifies the resonance circle that intersects regions of steepest perpendicular gradients in the EDF, thereby yielding the strongest amplification.

These relations allow qualitative analysis into how different EDFs drive the CMI. For a shell-type distribution—such as the shell (fig. S8A) or horseshoe (fig. S8B)—maximum growth occurs when the resonance circle is centered at the origin, $\left[v_{∥},v_{⊥}\right]=\left[v_{0},0\right]=\left[0,0\right]$, such that the resonance circle lies precisely along the edge of the shell distribution, maximizing the integral over the positive phase space gradient. Therefore, $v_{0}=\frac{k_{∥}c^{2}}{\omega_{\text{ce}}}=0$, implying $k_{∥}=0$, and thus, emission generated will propagate perpendicularly to the background magnetic field. The resonance radius $v_{r}$ in this case is equal to the velocity of the resonant electrons $v_{\text{res}}$ (see fig. S8A). Given that $v_{0}=0$ and $v_{r}=\sqrt{v_{0}^{2}-2c^{2}∆\omega }>0$, the condition $∆\omega =\frac{\omega -\omega_{\text{ce}}}{\omega_{\text{ce}}}<0$ must be satisfied, implying that $\omega <\omega_{\text{ce}}$. Furthermore, the greater the magnitude of ∆ω, the higher the resonant electron velocity $v_{\text{res}}=v_{r}$ will be (hence, higher electron kinetic energy). This implies that, observationally, the deviation between the wave frequency and the electron cyclotron frequency can serve as an indicator of the source electron energies. The corresponding kinetic energy of shell electrons that are in resonance with the wave can then be calculated in the relativistic form $E=\left(\Gamma -1\right)m_{e}c^{2}$, with the velocity of the electrons embedded in the Lorenz factor, and this leads to (combining $v_{\text{res}}=v_{r}=\sqrt{v_{0}^{2}-2c^{2}∆\omega }$ and $∆\omega =\frac{\omega -\omega_{\text{ce}}}{\omega_{\text{ce}}}$)

$$E_{\text{shell}}\approx 511\left(\frac{\omega_{\text{ce}}}{\omega }-1\right)\;\left(\text{in}\;\text{keV}\right)$$ (1)

Alternatively, a nonrelativistic approximation can be used, $E=\frac{1}{2}m_{e}v_{\text{res}}^{2}$, which yields $E_{\text{shell}}=\frac{511}{2}\left(1-\frac{\omega^{2}}{\omega_{\text{ce}}^{2}}\right)$ (in keV), in agreement with the expression provided previously (*72*). The relativistic and nonrelativistic formulas produce nearly identical results when the resonant electron energies are in the several kilo–electron volt range.

In contrast, for a loss-cone distribution (e.g., fig. S8C for loss-cone distribution and fig. S8D for conic distribution), maximum growth is achieved when the resonance circle is tangential to the edge of the loss cone (or the conic), where the perpendicular gradient of the EDF peaks. In this scenario, the resonance circle is no longer centered at zero ($v_{0}=\frac{k_{∥}c^{2}}{\omega_{\text{ce}}}\neq 0$), implying that $k_{∥}\neq 0$, and the resulting emissions are oblique.

By considering that the pitch angle of the resonant electrons should match the loss-cone angle, the parallel wave vector can be derived and is given by $k_{∥}=\frac{v_{0}\omega_{\text{ce}}}{c^{2}}$, and the resonance frequency simplifies to (*71*, *72*): $\omega \approx \frac{\omega_{\text{ce}}}{\sqrt{1-\frac{v^{2}}{c^{2}}}}$. As a result, the wave propagation is no longer fixed to the perpendicular direction, and the excited wave frequency will lie slightly above $\omega_{\text{ce}}$, as v≪c. The resonant energy of the electrons for loss cone–type EDF can be estimated from the geometry relation between $v_{\text{res}}$, $v_{r}$, and $v_{0}$, as shown in fig. S8C as $v_{\text{res}}^{2}=v_{0}^{2}-v_{r}^{2}=2c^{2}∆\omega$. Then, the relativistic energy of these electrons can be calculated as follows

$$E_{\text{losscone}}=\left(\Gamma -1\right)m_{e}c^{2}\approx 511\left(\frac{1}{\sqrt{3-2\frac{\omega }{\omega_{\text{ce}}}}}-1\right)\;\left(\text{in}\;\text{keV}\right)$$ (2)

Alternatively, a nonrelativistic approximation can be also used, $E=\frac{1}{2}m_{e}v_{\text{res}}^{2}$, which can be derived from the simplified relation $\omega \approx \frac{\omega_{\text{ce}}}{\sqrt{1-\frac{v^{2}}{c^{2}}}}$. This leads to the expression for the loss-cone case: $E_{\text{losscone}}=\frac{511}{2}\left(1-\frac{\omega_{\text{ce}}^{2}}{\omega^{2}}\right)$ (in keV) as used previously (*72*). Notably, the relativistic and nonrelativistic expressions yield very similar results when the resonant electron energy is in the range of several kilo–electron volts.

These relations provide a framework for identifying AKR source crossings. AKR is primarily generated by the so-called horseshoe electron distribution (*17*, *77*), which combines features of both shell and loss-cone populations (fig. S8B). Observationally, AKR emissions are almost always detected at frequencies below *fce* during source crossings, consistent with predictions for shell-type distributions. However, contributions from loss cone–driven emissions slightly above *fce* cannot be entirely excluded. Such emissions could also result from waves propagated from nearby sources rather than being locally generated. Without growth-rate estimates from in situ particle measurements, it is not possible to distinguish these scenarios unambiguously. Given that these emissions may still contain information relevant to possible loss cone–driven generation, we retained them in our analysis.

In practice, source crossings are identified using wave magnetic field spectrograms from the Polar PWI instrument, which provide superior signal-to-noise ratio compared to electric field measurements. Electric field spectrograms are also used as supplementary constraints to help exclude spurious or unwanted signals. In the AKR source region, the instrument’s frequency resolution at ~200 kHz is ~3% of the local *fce*, implying that the frequency of an identified AKR source carries an uncertainty of about ±1.5% within a single frequency channel.

As noted earlier, larger wave frequency deviations from *fce* may indicate higher-energy resonant electrons. However, as illustrated in Fig. 1, CMI-generated emissions typically exhibit a sharp cutoff near *fce*, particularly when the spacecraft traverses the source region. In contrast, Polar frequently detects emissions extending well below *fce* without any visible cutoff, suggesting that such signals are not source crossings. These emissions may instead originate from backward-propagating L-O mode waves generated at higher altitudes or Z mode waves confined just below *fce*, adding complexity to automated detection efforts.

To address this, we conservatively define potential source regions as emissions exhibiting cutoff features near *fce* and with their lowest frequency between 96.5 and 101% of the local *fce*. The upper bound of 101% corresponds to loss cone–driven CMI with a typical electron energy of ~5.2 keV, as estimated from Eq. 2. This threshold is also consistent with established criteria for identifying radio source crossings at Jupiter (*14*, *71*, *72*). The lower bound of 96.5% is chosen to (i) reduce contamination from L-O mode and Z mode emissions and (ii) allow for emissions that may extend across a full frequency bin, up to 3% below *fce*. The 96.5% *fce* frequency threshold corresponds to shell-driven CMI with electron energies of ~17.6 keV as estimated from Eq. 1. This 96.5% *fce* to 101% *fce* frequency range thus balances completeness with reliability while minimizing false positives in source identification.

The detection procedure involves several preprocessing steps to ensure the reliability of identified source crossings. First, magnetic field spectrogram pixels exhibiting anomalously high *E*/*cB* ratios—an indicator of the wave refractive index (where *E*/*cB* = 1/*n*, with *E* being the wave electric field amplitude, *B* the magnetic field amplitude, *c* the speed of light, and *n* the refractive index)—greater than 0.8 are removed. This criterion is based on visual inspection and is consistent with source region characteristics reported previously (*12*). Emissions with *E*/*cB* < 0.1 are also excluded, as they are typically associated with magnetic field saturation artifacts. In addition, weak signals with a magnetic spectral density below 10^−13^ nT^2^ Hz^−1^ are filtered out to eliminate background noise and ambiguous low-intensity emissions. Last, at each time step, emissions falling within the defined frequency range (96.5 to 101% of *fce*) are examined, and the lowest-frequency crossing is recorded as an AKR source. For each such event, the time, frequency, and average intensity from two adjacent frequency channels are extracted to characterize the event. The final detection results are manually inspected to eliminate false positives. Specifically, candidate events are excluded if they do not exhibit a clear cutoff feature at *fce* or if they are identified as backward-propagating L-O mode emissions or Z mode waves extending notably below *fce*. In addition, isolated detections that appear only once with no neighboring source crossings in time are also discarded, as they are unlikely to represent genuine AKR source regions.

### Estimating the standard deviation of the *fwave*/*fce* ratios

Given that the frequency resolution of Polar/PWI during the AKR source crossings is on the order of ~3% of the local *fce*, the derived *fwave*/*fce* ratios are therefore subject to large uncertainties. To provide a more quantitative bound on our results, we employed a Monte Carlo approach: Gaussian noise was added to the measured AKR source frequencies, and this process was repeated 1000 times to determine the distribution boundaries, defined here as the means ± three standard deviations for each histogram bin, as shown in Fig. 2C.

Specifically, the minimum and maximum source frequencies obtained were *fwave* = 91.223 kHz and *fwave* = 258.079 kHz, respectively. Across this range, the instrumental frequency resolution varies from ∆f/f=1.89% to ∆f/f=4.32%, implying that the uncertainty within a single frequency channel can be as large as ±2.16% (4.32/2). To quantify histogram uncertainties associated with the instrument’s finite frequency resolution, we used a Gaussian Monte Carlo resampling procedure. For each detected AKR source frequency $f_{i}$, we assigned an uncertainty equal to half of the frequency resolution, $\Delta f_{i}/2$. A random perturbation was then generated as follows

$$\varepsilon_{i}=\frac{\Delta f_{i}}{2}\times N\left(0,1\right)$$

where N(0,1) is a standard normal distribution (symmetric with respect to zero). A new realization of the source frequencies was obtained by adding this perturbation

$$f_{i}^{\left(k\right)}=f_{i}+\varepsilon_{i}$$

with the Monte Carlo process repeated 1000 times (*k* = 1 to 1000). This procedure produces 1000 resampled frequency distributions that reflect the full range of possible frequency values within the instrumental uncertainty. For each of these 1000 realizations, we constructed a histogram using the same bin edges as in Fig. 2C. The mean count in each bin over all realizations defines the plotted bin height, while the standard deviation provides the vertical error bar. These error bars therefore quantify uncertainties in histogram counts arising solely from the frequency-resolution limits of the instrument.

### Field-line tracing of AKR sources and the Polar spacecraft

Magnetic field lines from both the Polar spacecraft and detected AKR source locations were traced down to an ionospheric altitude of 130 km, corresponding to the typical emission height of auroral features observed by the Polar UVI instrument. Field-line tracing was performed using the Tsyganenko-1989 geomagnetic field model (*57*). This model was selected because it requires only the *Kp* index—continuously available during the source crossings—whereas the newer models [e.g., Tsyganenko-1996; (*78*)] require solar wind parameters, which are partly missing.

### Auroral images from Polar

Auroral images from the Polar UVI instrument were processed with dayside dayglow contamination removed following the method described by a previous work (*79*). The auroral images used were taken near 1700 Å at the Lyman-Birge-Hopfield molecular nitrogen band by the UVI instrument, which provided improved auroral observations resulting from minimal O_2_ absorption. This dataset was used to derive the average auroral oval distribution shown in Fig. 3.

Given that the UVI instrument was typically turned off during AKR source crossings, we instead used auroral images from the Polar VIS instrument for some of the figures, taken near the oxygen 130.4-nm emission band, which corresponds to an auroral emission altitude of ~300 km. Although this altitude is higher than the 130-km reference level used for magnetic field footprint mapping, the difference is minor and introduces negligible errors in the comparison of projected locations. Consequently, the AKR source footprints are directly overlaid on the VIS auroral images, as shown in Fig. 4.

### Clustering of electron spectra in the AKR source region

To investigate the characteristics of precipitating electrons at AKR source locations, we conducted a clustering analysis of the observed electron energy spectra. Initial classification was performed visually, identifying four distinct spectral types as discussed and shown in Figs. 1 and 4. To formalize this scheme, we applied *k*-means clustering to the AKR source electron spectra, initially dividing the dataset into six clusters. Two of these clusters, which showed only minor variations of a common spectral pattern, were merged into the four main categories on the basis of Euclidean distance from the average spectra.

Special attention was given to low-energy spectra, some of which were initially misclassified into the diffuse-type spectra. In particular, a subset of low-energy spectra with lower electron flux was erroneously assigned to the diffuse-type category. To address this, we reclassified such cases by requiring the presence of high-energy peaks (>1 keV) as a criterion to distinguish diffuse-type spectra from truly low-energy populations. Although the clustering is based solely on spectral shape and does not invoke physical definitions of broadband, monoenergetic, or diffuse precipitation (e.g., criteria based on flux or energy thresholding), it nevertheless provides a useful empirical framework for comparing electron spectral types across AKR source regions.

A previous study (*35*) has shown that more than 60% of auroral electron spectra exhibit features associated with multiple acceleration mechanisms on the basis of long-term observations from the FAST and Defense Meteorological Satellite Program satellites. The Polar dataset used in this study lacks pitch-angle information and has limited spectral resolution, making it insufficient for resolving such detailed acceleration processes. Nevertheless, the energy bandwidths observed for the monoenergetic and broadband categories are broadly consistent with acceleration by quasistatic potential drops and Alfvén waves, both of which produce characteristic spectral signatures. Low-energy precipitation may result from weak acceleration driven by either mechanism and therefore provides limited diagnostic information. Diffuse-type events are rare in our dataset, but their consistent occurrence near the equatorward boundary of the auroral oval lends confidence to the classification results.

### Identification of discrete aurora and extraction of auroral parameters

To distinguish discrete regions in diffuse auroral features in VIS images and to provide a quantitative measure of auroral features, we performed a clustering analysis on the basis of three physically motivated image features: (i) local auroral intensity, (ii) local standard deviation of intensity, and (iii) local intensity gradient. These features respectively capture emission brightness, small-scale spatial variability, and sharpness of auroral boundaries, collectively providing an objective measure of auroral structuring. Discrete auroras exhibit clear boundaries, whereas diffuse auroras appear as smooth background emissions or are too faint for reliable detection, as illustrated in Figs. 4 and 5.

Before feature extraction, Polar VIS images were preprocessed to enhance auroral structures and reduce background noise. First, calibrated auroral intensities were transformed logarithmically to compress the dynamic range and emphasize faint features, with a small offset added to handle zero values [$I\to {\text{log}}_{10}\left(I+\epsilon \right),\epsilon =1$]. The transformed intensities were then normalized to [0, 1]. Local spatial statistics were computed using a 5 by 5–pixel neighborhood. The local standard deviation was computed over a 5 by 5–pixel neighborhood as the square root of the local variance, which quantifies small-scale spatial variability. The intensity gradient magnitude was computed from horizontal and vertical gradients to quantify boundary sharpness. For each VIS image, pixels within a circular region of ~1° MLAT radius centered on the spacecraft footprint were transformed into a three-dimensional feature vector

X=[Intensity,Standard deviation,Gradient of intensity]

This feature set captures brightness, spatial variability, and boundary sharpness, forming the basis for subsequent classification. *k*-Means clustering was applied to separate discrete and diffuse aurora pixels. This method does not rely on predefined thresholds but identifies optimal partitions in the three-dimensional feature space, objectively distinguishing structured (bright, high-variance, high-gradient) aurora from diffuse (dim, low-variance, low-gradient) aurora. To refine the classification, clustering results were manually adjusted to yield three to five clusters, with the optimal separation of discrete aurora pixels selected via visual inspection. The resulting auroral detection is illustrated in Fig. 5 (A and B). An example of such clustering and selection of cluster is shown in fig. S9.
