# Supplementary material for: A radio oval above Earth’s auroral oval
Source: Sci Adv. 2026 Mar 27;12(13):eaec4114. doi: 10.1126/sciadv.aec4114 (PMC13025032; doi:10.1126/sciadv.aec4114)
Supplement: Supplementary file 1 — Figs. S1 to S9 [file sciadv.aec4114_sm.pdf]

Science Advances
AAAS

Supplementary Materials for

A radio oval above Earth's auroral oval

Siyuan Wu *et al.*

Corresponding author: Siyuan Wu, wusiyuan826@gmail.com

Sci. Adv. **12**, eaec4114 (2026)
DOI: 10.1126/sciadv.aec4114

This PDF file includes:

Figs. S1 to S9

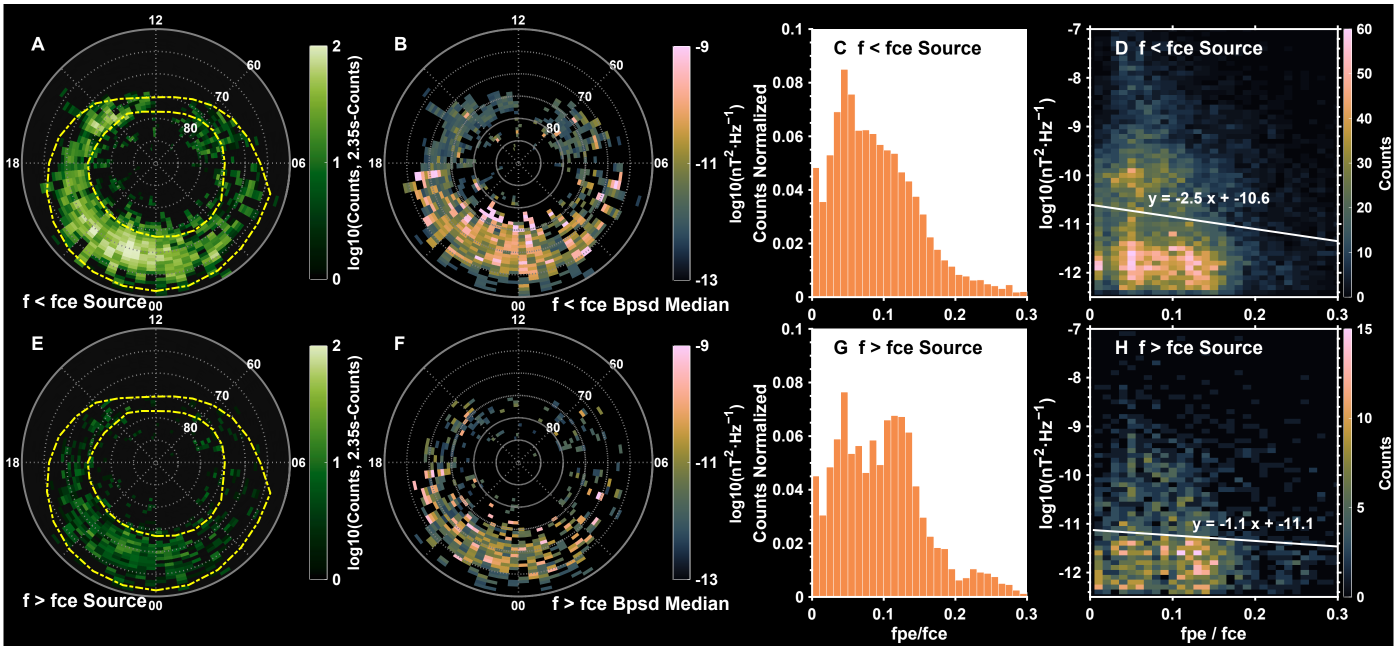

Fig. S1. Comparison of AKR radio ovals for *fwave > fce* and *fwave < fce* cases. A–D, Results for AKR sources with *fwave<fce*: A, footprints of AKR sources projected to auroral altitudes, in the same format as Fig. 3. B, median AKR wave intensity distribution, color-coded by magnetic wave intensity. C, *fpe/fce* ratio at the AKR source regions. D, Number of AKR sources as a function of the fpe/*fce* ratio and wave intensity. The white line represents a simple linear fit. A clear negative correlation is observed, indicating that stronger AKR sources tend to occur at smaller *fpe/fce* ratios. E-H, same as A–D, but for AKR sources with *fwave>fce*.

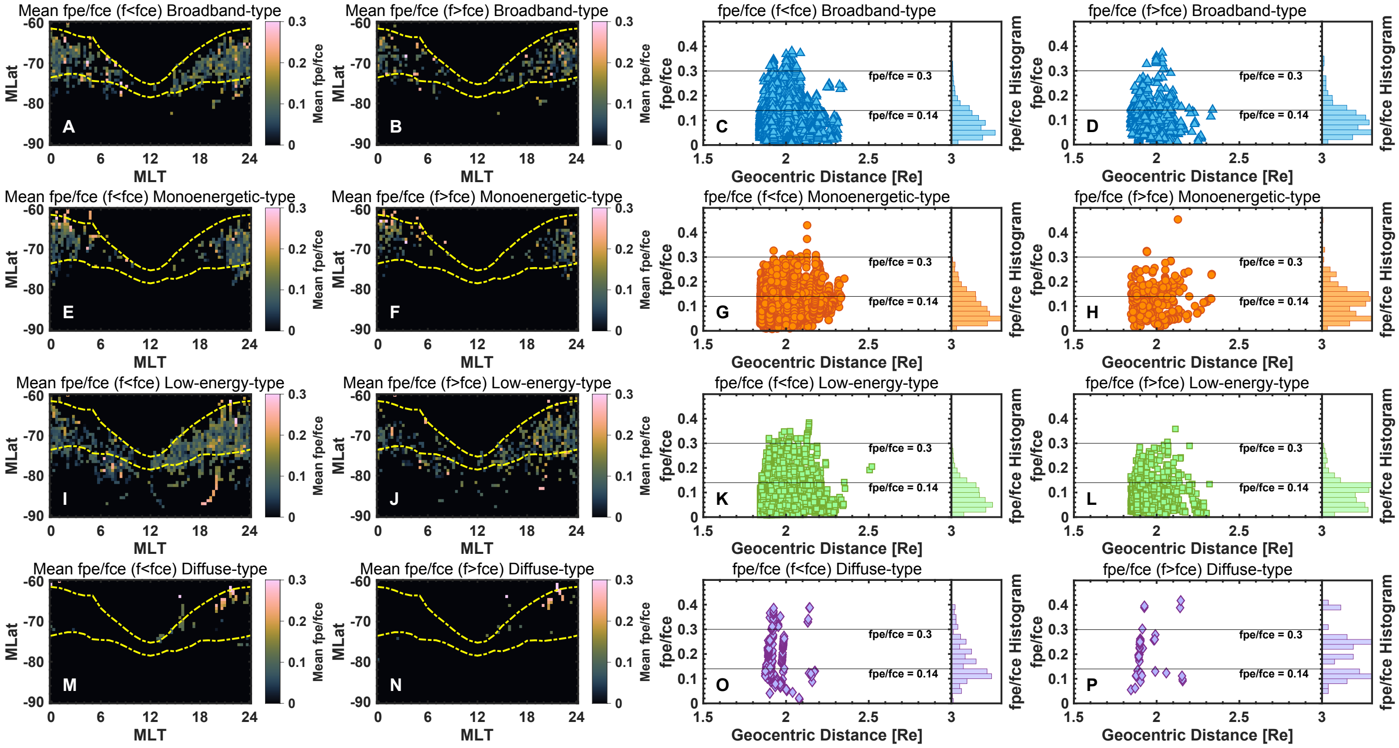

Fig. S2. *fpe/fce* ratios for different types of electron spectra. A–D, *fpe/fce* ratios for broadband-type spectra associated with AKR sources. A, Mean *fpe/fce* ratio in AKR source regions where *fwave < fce*, shown as a function of magnetic local time (MLT) and magnetic latitude (MLat). B, Same as A, but for AKR sources where *fwave > fce*. C, *fpe/fce* ratio for *fwave < fce* broadband sources as a function of geocentric distance. The side box gives the normalized distribution histogram of the *fpe/fce* ratio. D, Same as C, but for *fwave > fce* broadband sources. E–H, Same

format as panels A–D, but for AKR sources associated with monoenergetic-type spectra. I–L, Same format as above, but for AKR sources associated with low-energy-type spectra. M–P, Same format, but for AKR sources associated with diffuse-type spectra.

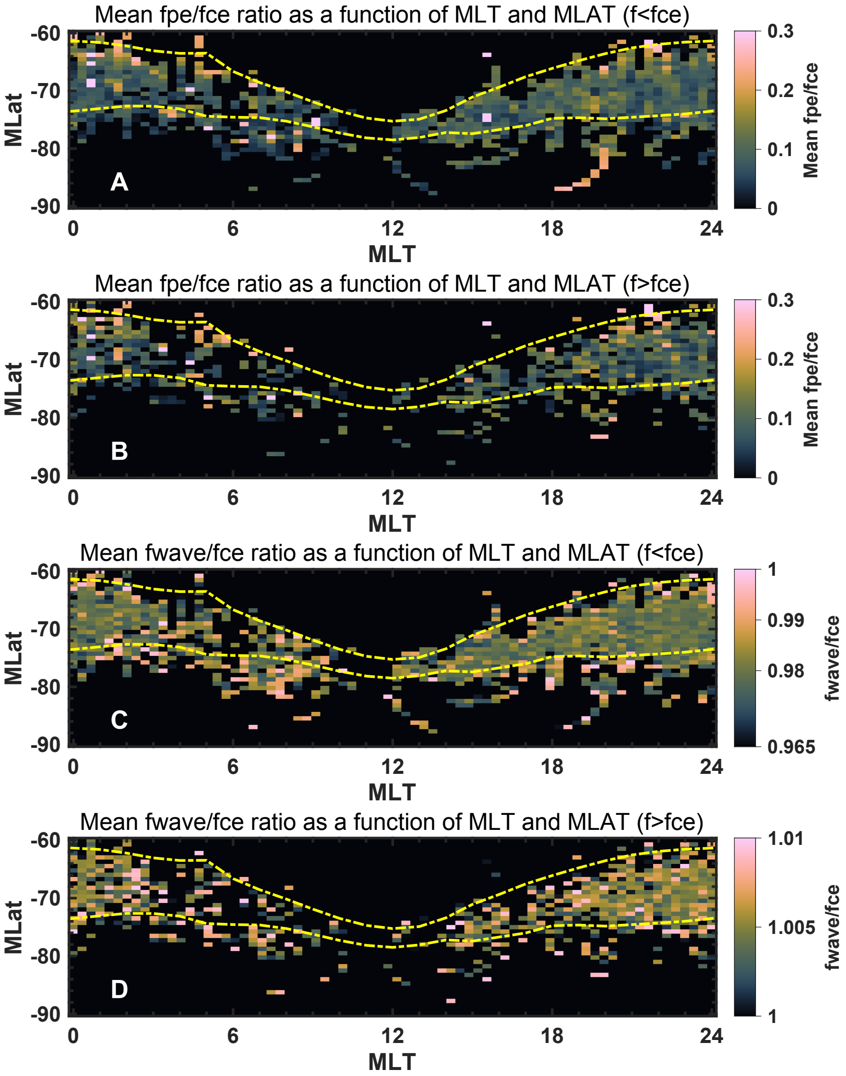

Fig. S3. ***fpe/fce*** **and fwave/*****fce*** **ratios for AKR sources with** ***fwave < fce*** **and** ***fwave > fce*****.** A, Mean *fpe/fce* ratios for AKR sources with *fwave < fce*, plotted as a function of MLT and MLAT. B, Same as A but for AKR sources with *fwave > fce*. C, Mean fwave/*fce* ratios for f*wave < fce* sources, shown as a function of MLT and MLAT. D, Same as C but for *fwave > fce* sources. The yellow contours indicate the auroral oval boundaries, identical to those in Fig. 3.

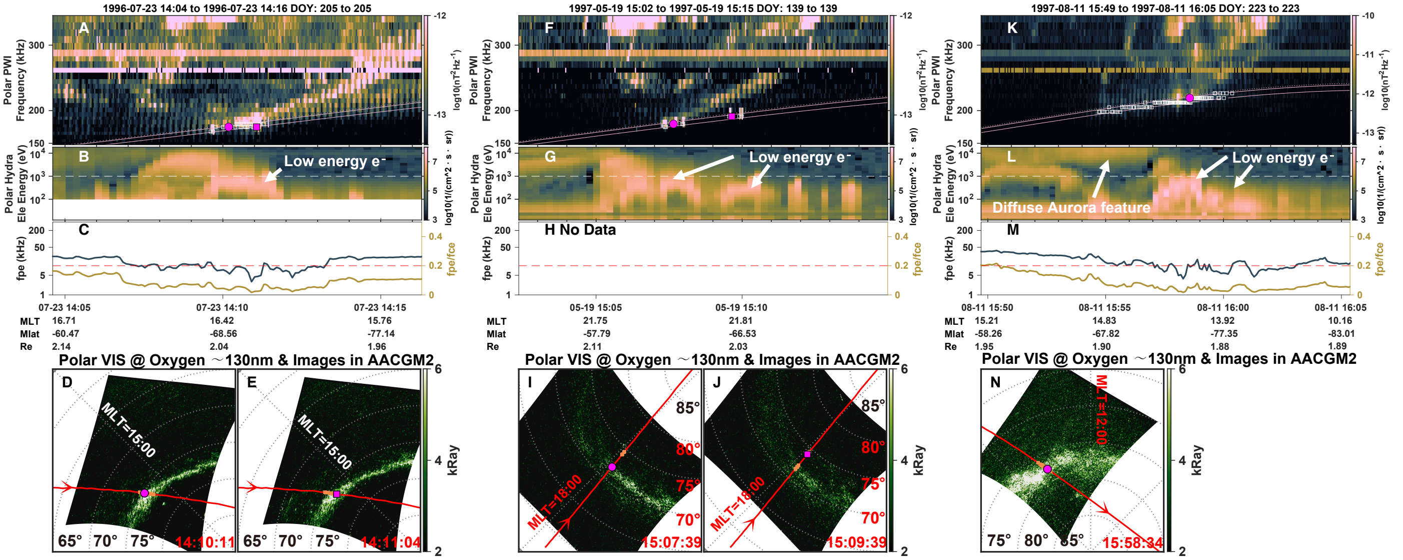

Fig. S4. Three additional examples of AKR sources associated with low-energy electron spectra. A, Spectrogram of the wave magnetic component measured by the Polar PWI instrument, with wave intensity color-coded as a function of time and frequency. B, Omni-directional electron differential energy flux measured by the Hydra instrument. C, Simultaneous measurements of fpe and *fpe/fce* ratios. D–E, Polar VIS auroral images near the time of source crossing. F–J and K–N, Two additional AKR source examples.

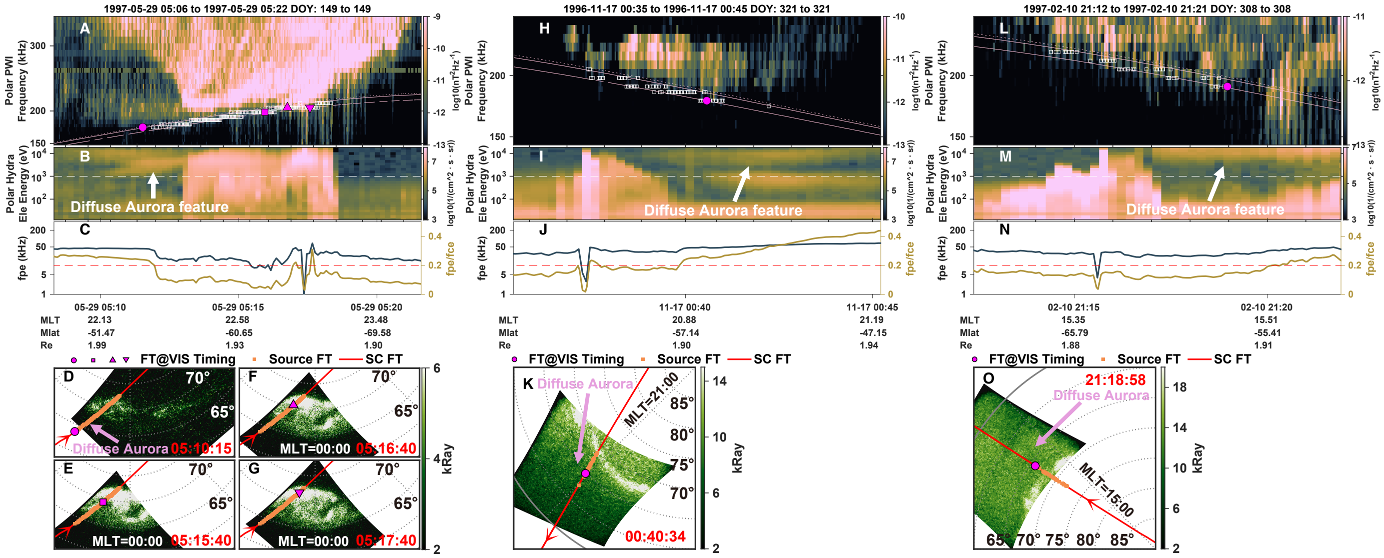

Fig. S5. Three additional examples of AKR sources located above diffuse aurora, shown in the same format as Fig. 4 and Fig. S4.

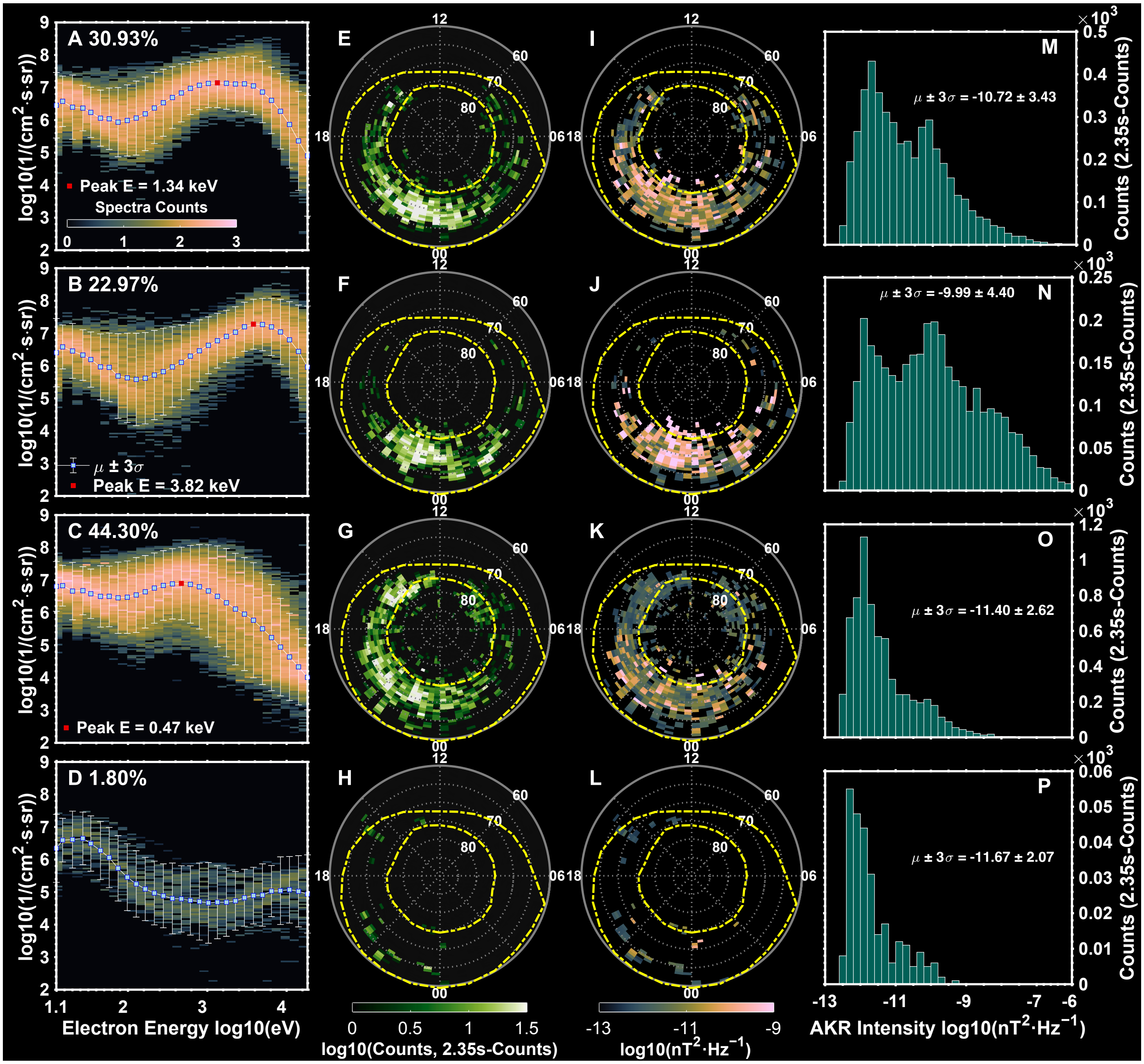

Fig. S6. Statistical characteristics of the four types of electron spectra and their associated AKR radio oval distributions, in the same format as Fig. 5, but for AKR sources with *fwave* < *fce* only.

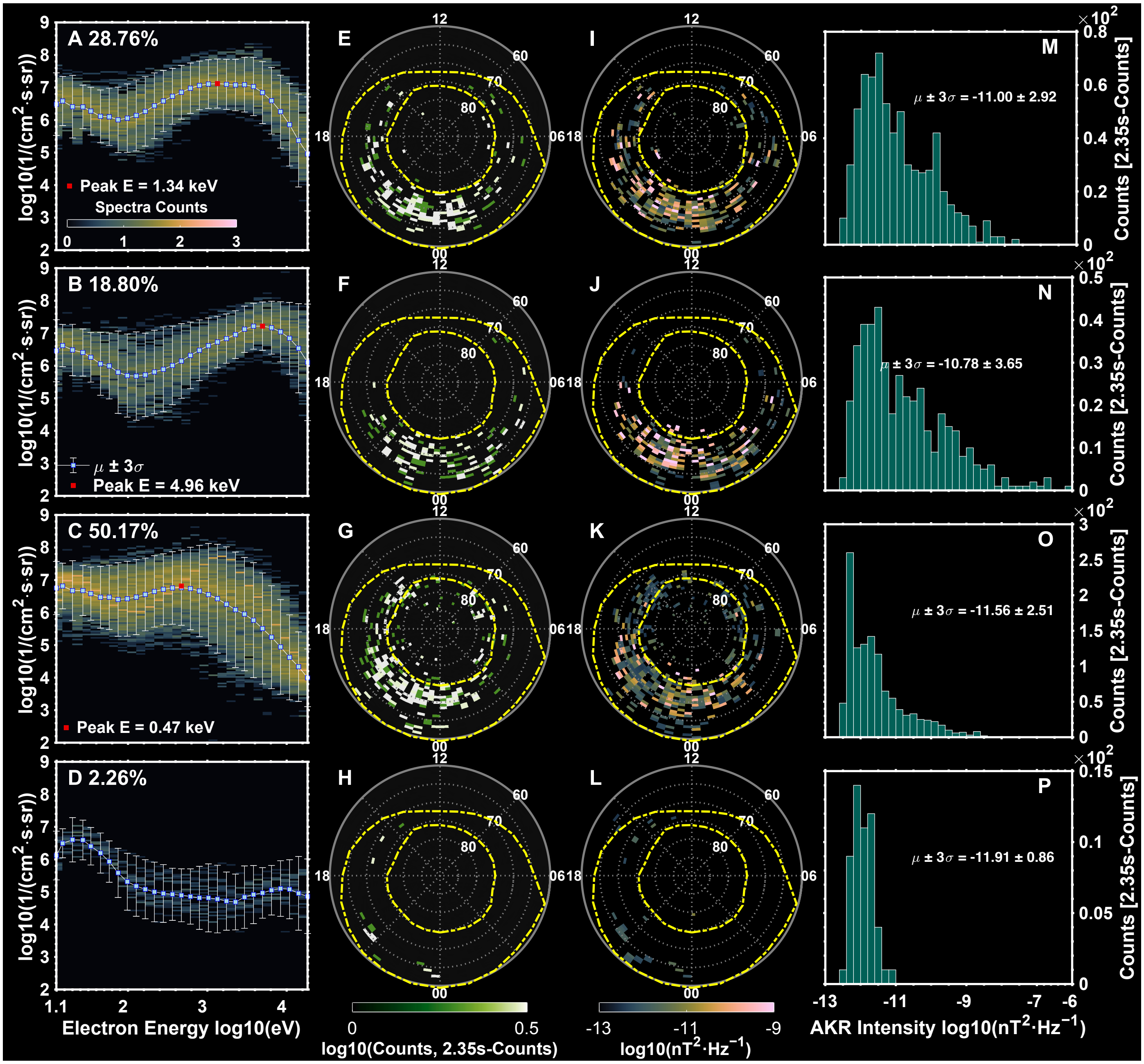

Fig. S7. Statistical characteristics of the four types of electron spectra and their associated AKR radio oval distributions, in the same format as Fig. 5, but for AKR sources with *fwave* > *fce* only.

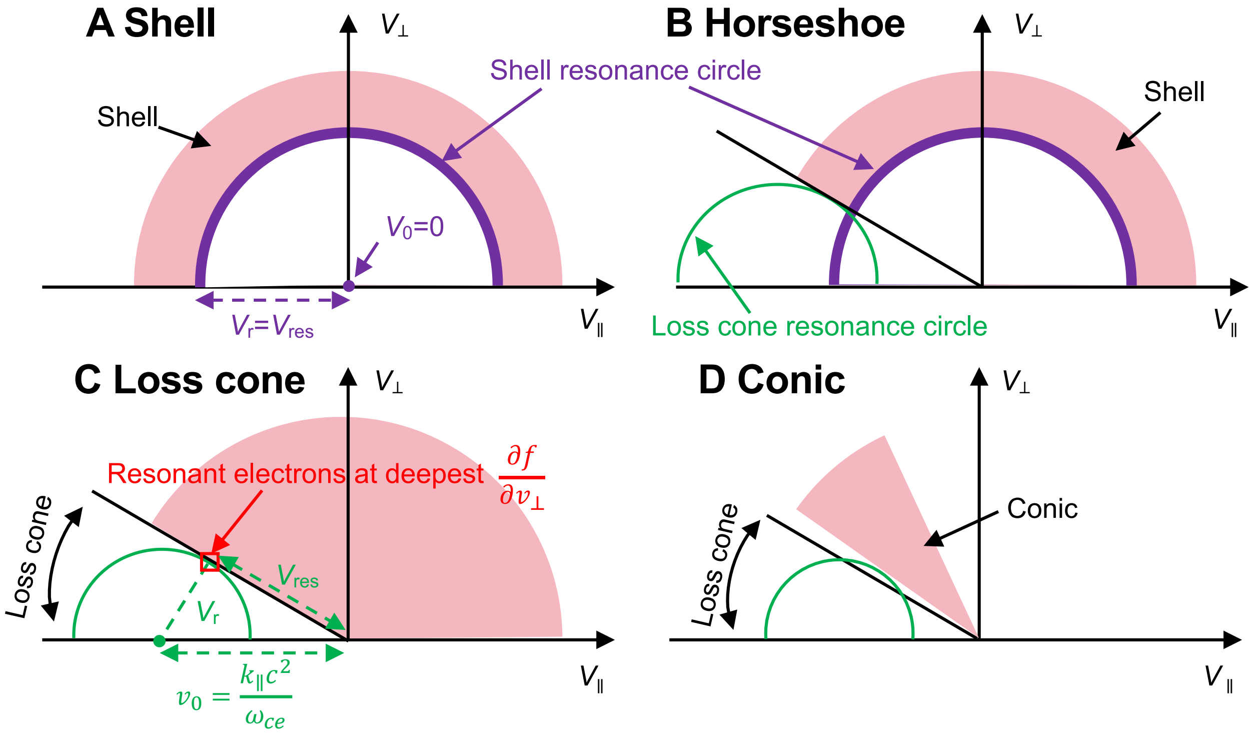

Fig. S8. Illustration of the resonance circle and the unstable EDFs. A. Shell electron distribution (red-shaded region). The cyclotron resonance circle (purple) is centered at the origin. The center of the resonance circle is given by $v_0 = \frac{k_\parallel c^2}{\omega_{ce}}$, and the radius by $v_r = \sqrt{v_0^2 - 2c^2\Delta\omega}$, where $\Delta\omega = \frac{\omega - \omega_{ce}}{\omega_{ce}}$. The velocity of resonant electron is denoted by v_{res}. In the shell case, the resonance circle is centered at the origin, $v_{res} = v_r$. B. Horseshoe distribution (red-shaded region), comprising a partial shell and a loss cone. Both the shell resonance circle (purple) and the loss cone resonance circle (green) intersect the electron density gradients. However, the shell resonance circle contributes more strongly to wave growth due to its greater overlap with the shell electron population. C. Loss cone distribution (red-shaded region). D. Electron conic distribution (red-shaded region), with a resonance circle similar to that of the loss cone case.

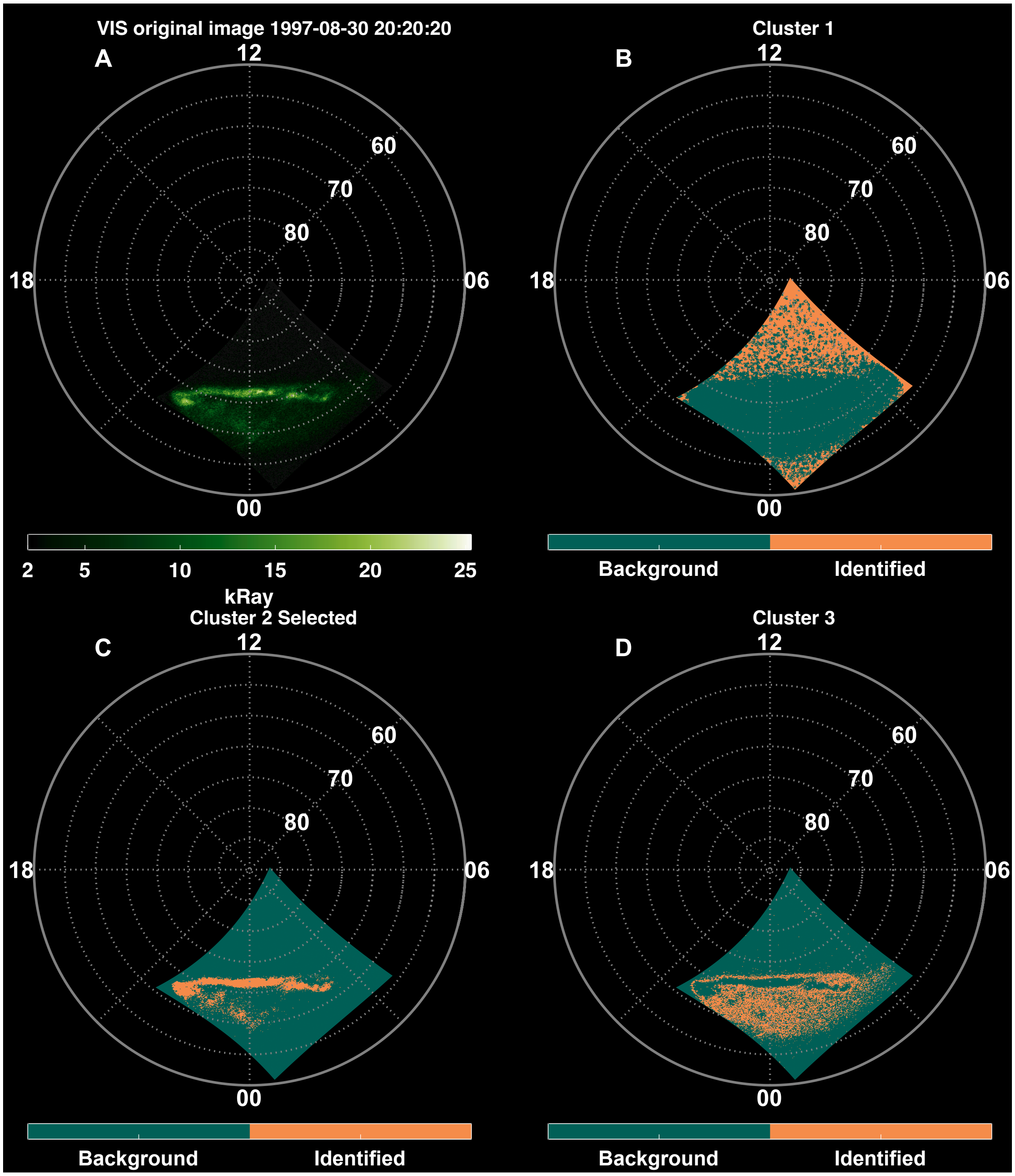

Fig. S9. Example of auroral clustering from Polar VIS images. A, Polar VIS image taken on 1997-08-30 at 20:20:20 UT. B, Output of Cluster 1 from the algorithm, with pixels assigned to this cluster highlighted in orange. C, Output of Cluster 2, shown in orange; this cluster is selected as the discrete-aurora category. D, Output of Cluster 3, with identified pixels highlighted in orange.
